# THBS1^+^ macrophages fuel pathogenic inflammation in alcohol-associated liver disease

**DOI:** 10.1097/HC9.0000000000001046

**Published:** 2026-09-18

**Authors:** Wentao Xu, Shanshan Wu, Jia Gao, Chen Cheng, Conghan Li, Shaocheng Hong, Jianshang Huang, Jinjing Zhang, Chenchen An, Miaomiao Wu, Xiang Li, Xin Wang, Yan Zhang, Hua Wang, Derun Kong, Wei He

**Affiliations:** 1 Department of Oncology, The First Affiliated Hospital of Anhui Medical University, Hefei, China; 2 Innovation and Entrepreneurship Laboratory for College Students, Anhui Medical University, Hefei, China; 3 Department of Gastroenterology, The First Affiliated Hospital of Anhui Medical University, Hefei, China; 4 School of Pharmaceutical Sciences, Anhui Medical University, Hefei, China; 5 Department of General Surgery, The Second Hospital of Anhui Medical University, Hefei, China; 6 Molecular Biology, Department of Biochemistry, Anhui Medical University, Hefei, China; 7 Laboratory Animal Platform, Institute of Artificial Intelligence, Hefei Comprehensive National Science Center; 8 Department of Infectious Diseases, The First Affiliated Hospital of Anhui Medical University, Hefei, China

**Keywords:** alcohol-induced injury, cell differentiation, inflammation, mononuclear phagocytes, single-cell RNA sequencing

## Abstract

**Background:**

Alcohol-associated liver disease (ALD) is one of the most common chronic liver diseases worldwide, with a pathological spectrum ranging from simple steatosis to steatohepatitis and, ultimately, cirrhosis. Severe alcohol-associated hepatitis (sAH) arises acutely within this continuum as a rapidly developing inflammatory condition associated with high mortality and poor prognosis.

**Methods:**

To investigate important cellular subpopulations, we integrated single-cell RNA sequencing (scRNA-seq) data from the peripheral blood and liver tissues of healthy individuals and patients with ALD (including those with alcoholic cirrhosis and sAH). A murine metabolic dysfunction–associated steatotic liver disease and alcohol-associated liver disease (MetALD)-like model was established by combining a metabolic dysfunction–associated steatohepatitis–inducing diet with controlled ethanol exposure. The model was used for histopathological analyses, and CD45^+^ cells from peripheral blood and liver tissues were isolated for scRNA-seq. Analyses included cell composition profiling, differential expression, pathway enrichment, pseudotime trajectory inference, transcriptional regulatory network reconstruction, and cell-cell communication analysis. In parallel, we generated THBS1‑KO and IL15‑KO immortalized bone marrow-derived macrophages cell lines in vitro and conducted functional assays to validate their roles.

**Results:**

ScRNA-seq data revealed significant enrichment of IL15^+^ monocytes and THBS1^+^​​​​ monocyte-derived macrophages (moMFs) in patients with sAH, both exhibiting highly proinflammatory characteristics. Pseudotime analysis indicated that circulating IL15^+^ monocytes could dynamically differentiate into hepatic THBS1^+^ moMFs. Pathological staining and scRNA-seq data from the MetALD mouse model yielded similar results, further confirming the dynamic evolution of IL15^+^​​​​ monocytes into THBS1^+^​​​​ moMFs. In vitro cellular experiments also substantiated the proinflammatory roles of THBS1 and IL15.

**Conclusions:**

Our study reveals that circulating IL15^+^​​​​ monocytes dynamically differentiate into hepatic THBS1^+^​​​​ moMFs, and the combined proinflammatory action of these subsets accelerates the progression of ALD.

## INTRODUCTION

Alcohol-associated liver disease (ALD) is a multifactorial disorder caused by excessive chronic alcohol consumption. It spans a wide pathological spectrum, including steatosis, hepatitis, fibrosis, and cirrhosis, and is associated with an increased risk of HCC.^1^,^2^ ALD often coexists with conditions such as hepatitis B and C, obesity, metabolic syndrome, and diabetes, contributing to its complex pathophysiology.^3^,^4^ During disease progression, some patients develop acute liver injury manifesting as severe alcohol-associated hepatitis (sAH). As one of the most aggressive ALD phenotypes, sAH has a rapidly worsening clinical course and high short-term mortality, with over 30% of patients dying within 90 days.^5^,^6^,^7^

The complex pathophysiology of ALD, particularly in severe forms, such as sAH, is underscored by robust immune responses. This involves dynamic interactions between hepatic parenchymal and nonparenchymal cells, along with the extensive recruitment of peripheral immune cells to the liver.^8^ Among these, macrophages are a central component of the hepatic immune system and constitute ~80% of the total liver immune cells and play an indispensable role in the pathogenesis and progression of ALD.^9^

Hepatic macrophages can be divided into 2 major subpopulations based on their developmental origin: resident KCs and monocyte-derived macrophages (moMFs), which differentiate from bone marrow monocytes migrating to the liver.^10^ In sAH, the hepatic macrophage compartment undergoes significant remodeling, characterized by extensive macrophage infiltration within the liver, where resident KCs are largely replaced by a functionally heterogeneous population of moMFs.^11^ Single-cell RNA sequencing (scRNA-seq) has further identified various macrophage subpopulations in the liver of patients with sAH and alcoholic cirrhosis, including a unique C1Q^+^ macrophage subpopulation that may compensate for clearing dead cells but could also amplify inflammation and drive sAH progression.^11^ Similar conclusions were drawn from the western diet alcohol mouse model, in which KCs were replaced by functionally heterogeneous moMFs, and infiltrating monocytes/macrophages were predicted to have proinflammatory effects.^12^ This suggests that infiltrating macrophages play a crucial role in sAH, and their recruitment, differentiation, and maturation from monocytes into hepatic macrophages may involve the upregulation of transcription factors such as members of the C/EBP family and ATF3, which regulate macrophage differentiation and function.^13^,^14^ Furthermore, recent scRNA-seq and fate mapping analyses in metabolic dysfunction–associated steatohepatitis have revealed key regulatory signals, such as the Notch-RBPJ pathway, that control the differentiation of monocytes into specific macrophage subpopulations.^15^ However, the differentiation trajectories and underlying mechanisms of monocyte-macrophage lineages in ALD remain largely unknown.

This study integrated scRNA-seq data from whole-blood and liver tissues of healthy individuals and patients with ALD. Our findings revealed a significant enrichment of IL15^+^ monocytes and THBS1^+^ moMFs in patients with sAH, which were further confirmed to possess potent proinflammatory functions. Using multiple pseudotime trajectory analyses, we elucidated the dynamic differentiation trajectory of IL15^+^ monocytes from circulation to intra-tissue THBS1^+^ moMFs, and concurrently identified their key transcriptional regulatory networks. Notably, during this differentiation process, critical transcription factors such as the C/EBP family, ATF3, and STAT1 were significantly activated. Moreover, cell-fate mapping and coexpression profiling collectively identified CXCL8 as a key driver gene of this differentiation trajectory. ScRNA-seq and histopathological results from our established metabolic dysfunction–associated steatotic liver disease and alcohol-associated liver disease (MetALD) mouse model further supported this dynamic cellular alteration pattern, and in vitro experiments confirmed the pivotal roles of IL15 and THBS1 in amplifying inflammation.

## METHODS

The antibodies and primers used in this study are included in Supplemental Table, https://links.lww.com/HC9/C474. The detailed methods are given in Supplemental Methods, https://links.lww.com/HC9/C475.

### Data collection

We collected public data sets based on the following criteria: (1) Healthy Liver, (2) ALcoholic Cirrhosis (Liver and white blood cells [WBC]), and (3) Severe Alcohol Hepatitis (Liver and WBC). The scRNA-seq data sets GSE255772^16^ and GSE136103,^17^ as well as the snRNA-seq data set GSE256398,^18^ were retrieved from the GEO database. The Chip-seq data set GSM2845675 was retrieved from the GEO database.

### Statistical analysis

All statistical analyses and data visualizations were performed using R software or GraphPad Prism 10.1.2. Comparisons between 2 groups were conducted using either a 2-tailed unpaired/paired *t* test or a 2-tailed Mann-Whitney *U* test. Comparisons involving more than 2 groups were performed using either 1-way ANOVA with Tukey’s multiple comparisons test or 1-way ANOVA with the Dunnett multiple comparisons test. For differential expression analyses, *p* values were adjusted for multiple testing using the Benjamini-Hochberg false discovery rate correction. A *p* value or an adjusted *p* value <0.05 was considered statistically significant.

## RESULTS

### Multimodal characterization of hepatic and immune cell landscapes across ALD

**FIGURE 1:**
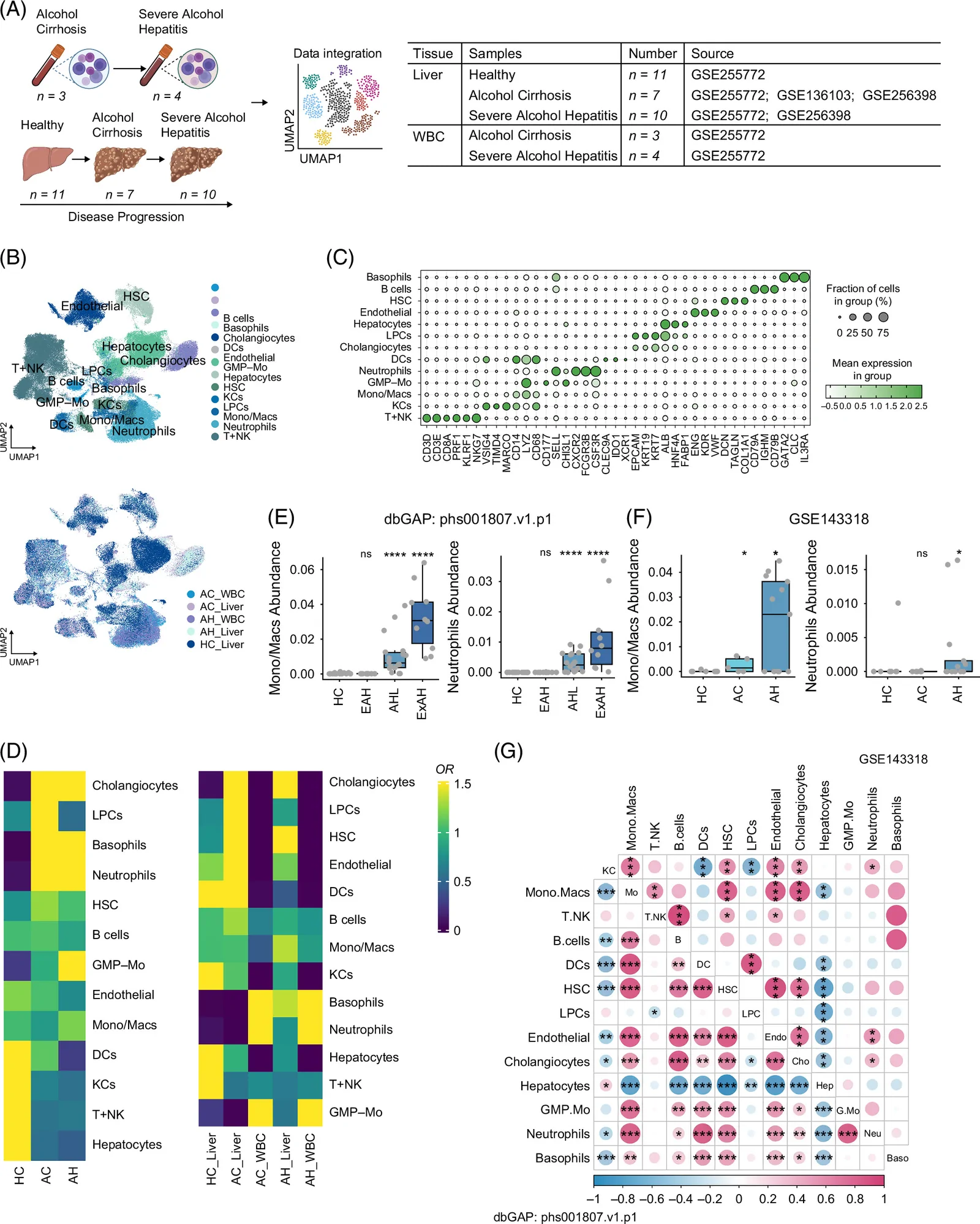
Multimodal characterization of hepatic and immune cell landscapes across ALD. (A) Data source schematic. (B) UMAP plots of different tissues and cell subtypes correlated by color. (C) Dot plot shows the key markers used to describe cluster identity and link it to cell type. (D) Group preference estimated by Ro/e score. (E) Cell‑type deconvolution of bulk RNA‑seq samples from the phs001807.v1.p1 cohort, showing inferred proportions of monocytes/macrophages (Mono/Macs) and neutrophils across HC, EAH, AHL, and ExAH. Each dot represents an individual sample. (F) Cell‑type deconvolution of bulk RNA‑seq samples from the GSE143318 cohort, showing inferred proportions of Mono/Macs and neutrophils across HC, AC, and AH. Each dot represents an individual sample. (G) Correlation matrix of cell type associations. *p<0.05, **p<0.01, ***p<0.001, ****p < 0.0001; ns, not significant. Abbreviations: AC, alcohol-associated cirrhosis; AH, alcoholic hepatitis; AHL, alcoholic hepatitis; ALD, alcohol-associated liver disease; EAH, early alcoholic hepatitis; ExAH, extended severe alcoholic hepatitis; HC, healthy controls; UMAP, Uniform manifold approximation and projection.

By integrating scRNA-seq data from 35 samples, we generated a comprehensive atlas of liver and peripheral blood from healthy controls, alcohol-associated cirrhosis, and sAH (Figure 1A). Uniform Manifold Approximation and Projection visualization of the merged data set revealed clear clustering of major cell populations (Figure 1B). Cell annotation identified expected hepatic and immune lineages, including hepatocytes, endothelial cells, cholangiocytes, HSCs, T and NK cells, B cells, basophils, KCs, moMFs, dendritic cells, and GMP Mo progenitors (Figure 1C).

Macrophage populations exhibited substantial remodeling across ALD stages. moMFs increased progressively, with the highest abundance observed in severe disease, consistent with enhanced monocyte recruitment and inflammatory differentiation. In contrast, KCs declined steadily, indicating depletion of resident macrophages and a transition from KCs to moMFs during ALD progression, consistent with previous reports^11^ (Figure 1D). Neutrophils increased markedly during ALD progression, consistent with previous studies supporting their critical role in alcoholic hepatitis.^16^ T/NK cells and B cells also exhibited substantial dynamic alterations, indicating broad remodeling of both innate and adaptive immune compartments throughout disease development.

To further validate these findings, we performed cell type deconvolution of 2 independent bulk RNA sequencing cohorts (phs001807.v1.p1 and GSE143318). Both data sets reproduced the macrophage remodeling pattern observed in the single-cell analysis (Figures 1E, F). Combined correlation analysis showed a coherent association structure across cohorts (Figure 1G), in which moMFs correlated positively with multiple inflammatory cell populations, whereas KCs displayed broad negative correlations. Together, these data indicate that moMFs play a prominent role in ALD.

### Poor prognosis of patients with alcoholic liver driven by myeloid cells

**FIGURE 2:**
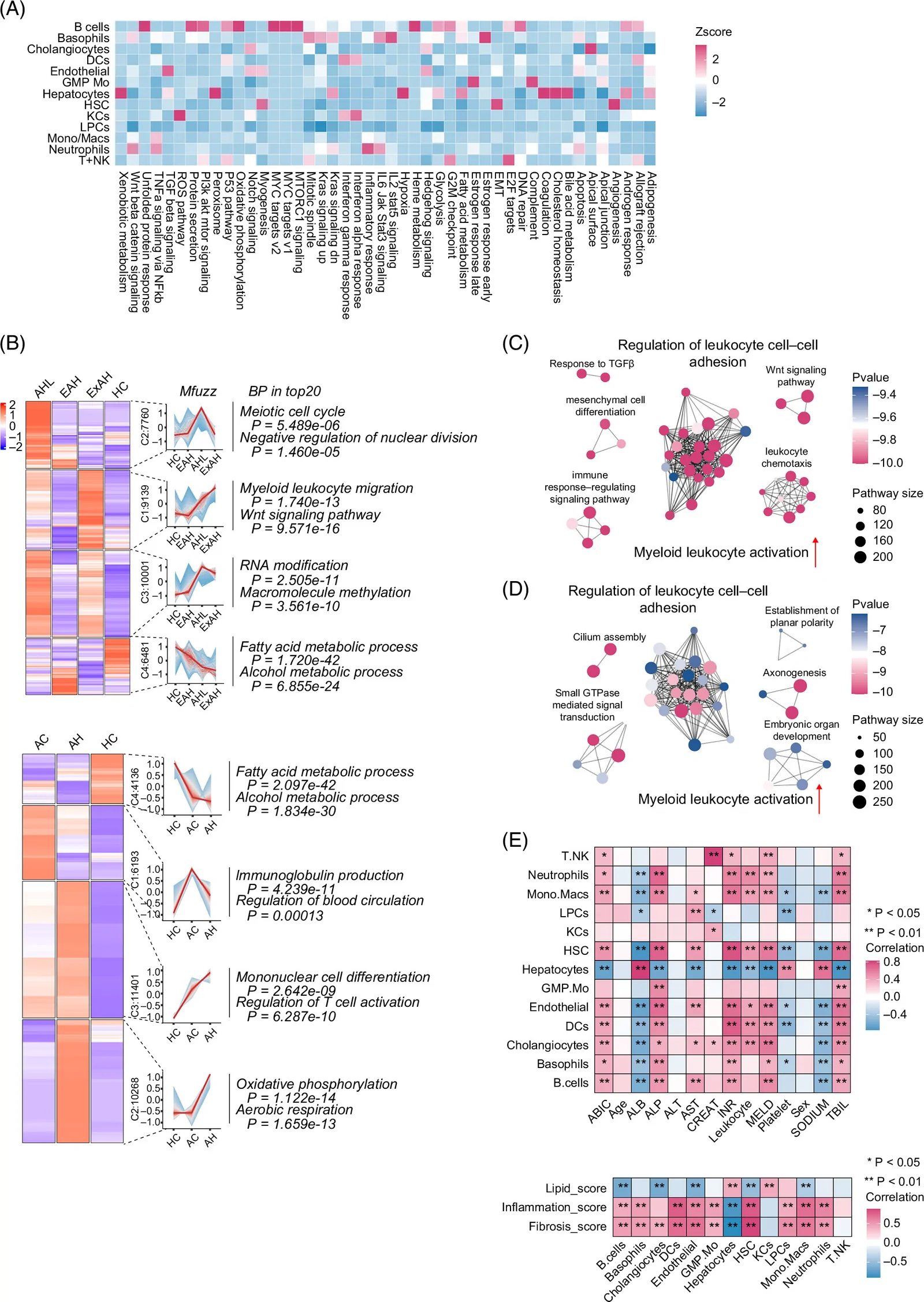
Poor prognosis of patients with alcoholic liver driven by myeloid cells. (A) Heatmap shows the enriched hallmarks in each cell type from all samples. (B) Heatmap representation of time-course RNA-seq data shows the rapid activation of typical myeloid-cell pathways across the progression from healthy controls to different stages of ALD. (C, D) Network of GO enrichment biological processes in the myeloid cell activation pathway in a heatmap. Node size represents gene number, edge thickness denotes overlap coefficient, and node color indicates −log10 (adjusted *p* value). (E) Correlation between cell proportion and clinical indexes after deconvolution. (F) Correlation between cell proportion and lipid deposition, inflammation, and fibrosis after deconvolution. *p<0.05, **p<0.01, ***p<0.001; ns, not significant. Abbreviations: ALD, alcohol-associated liver disease; GO, gene ontology.

Based on the HALLMARK gene set (Figure 2A), neutrophils exhibited pronounced activation of inflammation‑related pathways, including stands for tumor necrosis factor-alpha signaling, NF‑κB signaling, and interferon pathways. This pattern is consistent with the neutrophil‑driven amplification of inflammation reported in ALD by Guan et al.^16^ Meanwhile, moMFs displayed a broad and coordinated activation of functional pathways. In addition to the activation of classical inflammatory signaling such as IL6/JAK/STAT3 and stands for tumor necrosis factor-alpha/IL1, these cells also upregulated pathways related to migration, integrin‑mediated adhesion, phagocytosis, and antigen processing, indicating a highly responsive immunological state. This observation is further supported by recent findings from Guan et al.^16^ Furthermore, KCs showed significant enrichment of reactive oxygen species‑related pathways, indicating enhanced oxidative stress and a potential role in shaping the local inflammatory environment in ALD.

Through Mfuzz clustering analysis (Figure 2B), we observed that the progression of ALD follows a dual trajectory characterized by increasing immune activation and declining metabolic function. Modules related to inflammation and immune responses were progressively upregulated, and pathways linked to myeloid leukocyte migration and monocyte differentiation were consistently enhanced, reflecting the recruitment, maturation, and inflammatory reprogramming of myeloid cells. In contrast, metabolic modules showed a gradual reduction with disease severity, particularly those involved in alcohol metabolism and fatty acid metabolism, indicating a progressive impairment of ethanol clearance and lipid metabolic capacity.

In 2 independent cohorts (Figures 2C, D), enrichment network analyses demonstrated a highly consistent pattern of immune activation. Core-enriched pathways were centered on myeloid leukocyte activation, accompanied by enhanced leukocyte adhesion, chemotaxis, and migration signaling, forming a reproducible immunopathological signature across data sets. Correlation analysis (Figure 2E) further supported the clinical relevance of this myeloid immune axis. The abundances of moMFs and neutrophils were strongly positively correlated with disease severity indicators, including the Age‑bilirubin‑INR‑creatinine score, the MELD, AST, the international normalized ratio (INR), and total bilirubin, and were positively associated with both inflammation and fibrosis scores. The significant correlation between moMFs and platelet levels also suggests that interactions between inflammation and coagulation may contribute to the progression of ALD.

Taken together, these results delineate a core pathogenic trajectory of ALD characterized by progressive amplification of myeloid immune activation alongside a gradual decline in hepatic metabolic capacity. Moreover, the accumulation of neutrophils and moMFs showed strong correlations with clinical prognostic scores such as age‑bilirubin‑INR‑creatinine score and MELD, as well as with bilirubin levels, coagulation parameters, and fibrosis indices. These associations suggest that excessive activation of the myeloid immune axis not only reflects disease severity but may also actively drive adverse outcomes in ALD.

### IL15^+^ monocytes and THBS1^+^ moMFs are key contributors to poor outcomes in ALD

**FIGURE 3:**
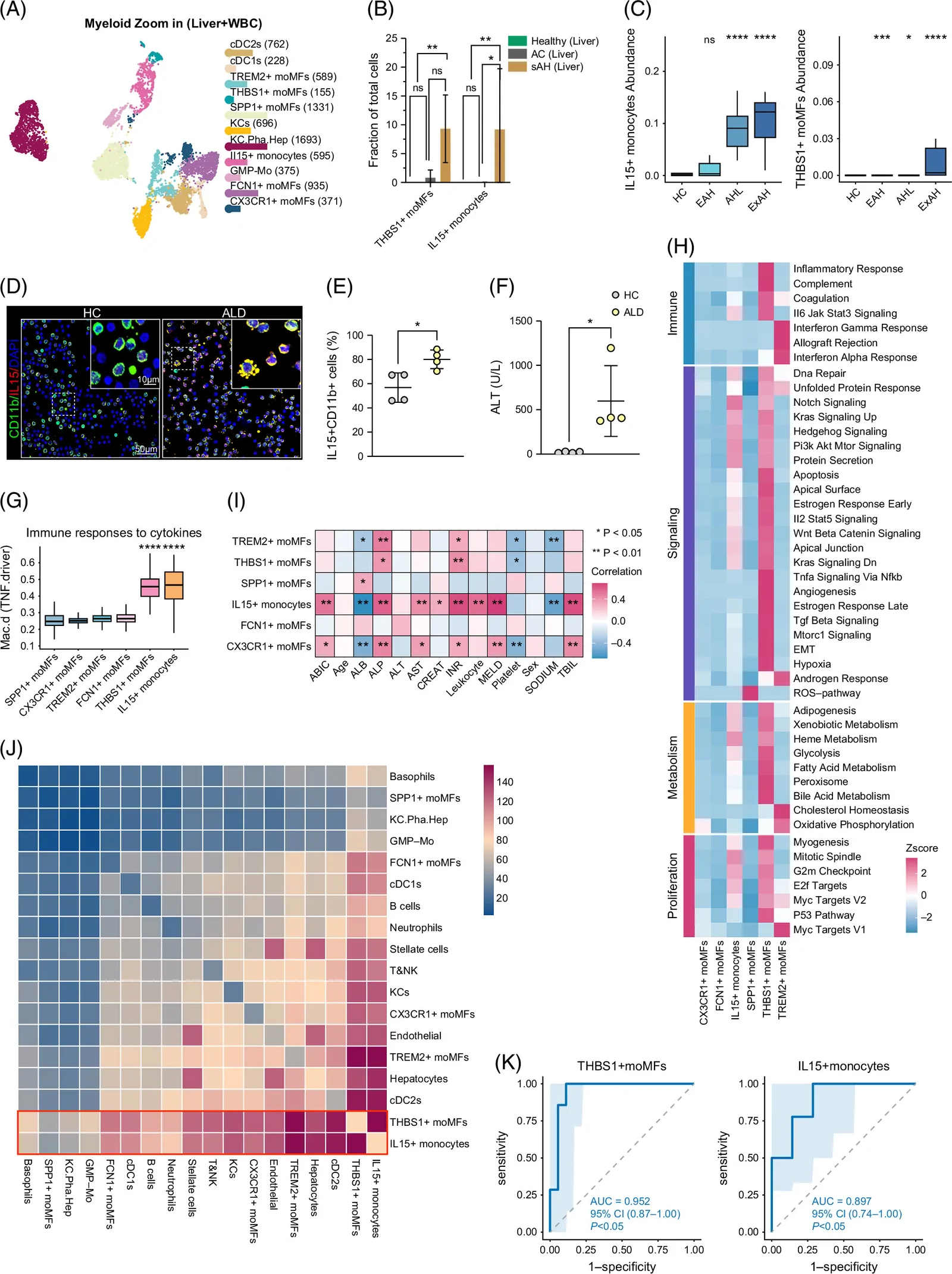
IL15^+^ monocytes and THBS1^+^ moMFs are key contributors to poor outcomes in ALD. (A) UMAP plots show clustering results of myeloid cells. (B) Quantification of cell cluster frequency representation among patients. Average and SEM are shown for each patient group. (C) Cell proportion histogram of IL15^+^ monocytes in RNA-seq data (left). Cell proportion histogram of THBS1^+^ moMFs in RNA-seq data (right). (D) Representative immunofluorescence staining of PBMCs from HC and patients with ALD, showing CD11b^+^ IL15^+^ cells. (E) Quantification of IL15^+^CD11b^+^ cells (%) in PBMCs from HC and patients with ALD. (F) Serum ALT levels in HC (n=4) and individuals with ALD (n=4). (G) Cytokines (TNF) drive diverse polarization states in myeloid cells. (H) Heatmap shows the enriched hallmarks in each myeloid cell subtype. (I) Correlation between myeloid cell proportion and clinical indexes. (J) Analysis of cell-cell interaction between myeloid subsets and other cells by CellPhoneDB. (K) ROC curves generated from the GSE143318 data set evaluating the diagnostic performance of THBS1^+^ moMFs and IL15^+^ monocytes. *p<0.05, **p<0.01, ***p<0.001; ns, not significant. Abbreviations: ALD, alcohol-associated liver disease; HC, healthy controls; moMF, monocyte-derived macrophage; PBMC, peripheral blood mononuclear cells; ROC, receiver operating characteristic; UMAP, uniform manifold approximation and projection.

Given the critical involvement of myeloid cells in ALD progression, we first extracted and reclustered myeloid cells from the liver and WBC. Based on canonical marker genes, we resolved multiple distinct myeloid subsets, including cDC2, cDC1, KCs, KC.Pha.Hep, GMP‑like cells, and several moMFs subsets such as TREM2^+^, THBS1^+^, SPP1^+^, FCN1^+^, and CX3CR1^+^ moMFs. Notably, one cluster displayed high IL15 expression together with monocyte-associated markers, supporting its annotation as IL15^+^ monocytes (Figure 3A; Supplemental Figures S1A, B, https://links.lww.com/HC9/C476).

We next assessed the distribution of these myeloid subsets across different stages of ALD severity. SPP1^+^ moMFs, THBS1^+^ moMFs, and IL15^+^ monocytes were selectively increased in patients with sAH (Figure 3B; Supplemental Figures S1C–E, https://links.lww.com/HC9/C476). Deconvolution of bulk RNA‑seq data sets further demonstrated that IL15^+^ monocytes were predominantly elevated in AHL and ExAH, whereas THBS1^+^ moMFs were increased across EAH, AHL, and ExAH (Figure 3C; Supplemental Figures S1F–I, https://links.lww.com/HC9/C476). Consistently, in bulk RNA-seq cohorts, IL15 and THBS1 exhibited differential expression across disease stages (Supplemental Figure S1J, https://links.lww.com/HC9/C476). To experimentally validate these findings, immunofluorescence staining of peripheral blood mononuclear cells from patients with ALD and HC confirmed a marked increase in CD11b^+^ IL15^+^ cells in ALD (Figures 3D–F).

Cytokine‑response profiling further demonstrated enhanced inflammatory activation across myeloid subsets, including IL15^+^ monocytes and THBS1^+^ moMFs (Figure 3G). Pathway enrichment analysis revealed that these 2 subsets were strongly enriched for inflammation‑related programs, while THBS1^+^ moMFs additionally displayed activation of extracellular matrix‑remodeling and fibrosis‑associated pathways (Figure 3H). These findings were supported by Kyoto Encyclopedia of Genes and Genomes and Gene ontology enrichment analyses (Supplemental Figures S2A–D, https://links.lww.com/HC9/C476), which consistently demonstrated enhanced activity in inflammatory response, extracellular matrix regulation, and collagen fibril organization pathways, underscoring the proinflammatory and profibrotic phenotypes of these 2 subsets (Supplemental Figures S2E–G, https://links.lww.com/HC9/C476). Correlation analysis further demonstrated that IL15^+^ monocytes and THBS1^+^ moMFs were closely associated with clinical indicators of hepatic injury and systemic inflammation (Figure 3I). Moreover, cell-cell interaction mapping showed that these subsets were among the most actively engaged populations within the hepatic immune microenvironment (Figure 3J), underscoring their involvement in inflammatory communication networks.

To assess clinical translational potential, receiver operating characteristic analysis in an independent bulk RNA-seq cohort showed that THBS1 and IL15 exhibited strong diagnostic performance for distinguishing sAH from controls, supporting their potential utility as diagnostic biomarkers (Figure 3K).

Together, these findings identify IL15^+^ monocytes and THBS1^+^ moMFs as key contributors to the inflammatory landscape of ALD. Their selective expansion in severe diseases, combined with strong activation of inflammatory and fibrogenic programs, underscores their potential roles in amplifying hepatic injury.

### IL15^+^ monocytes differentiate into THBS1^+^ moMFs during ALD progression

**FIGURE 4:**
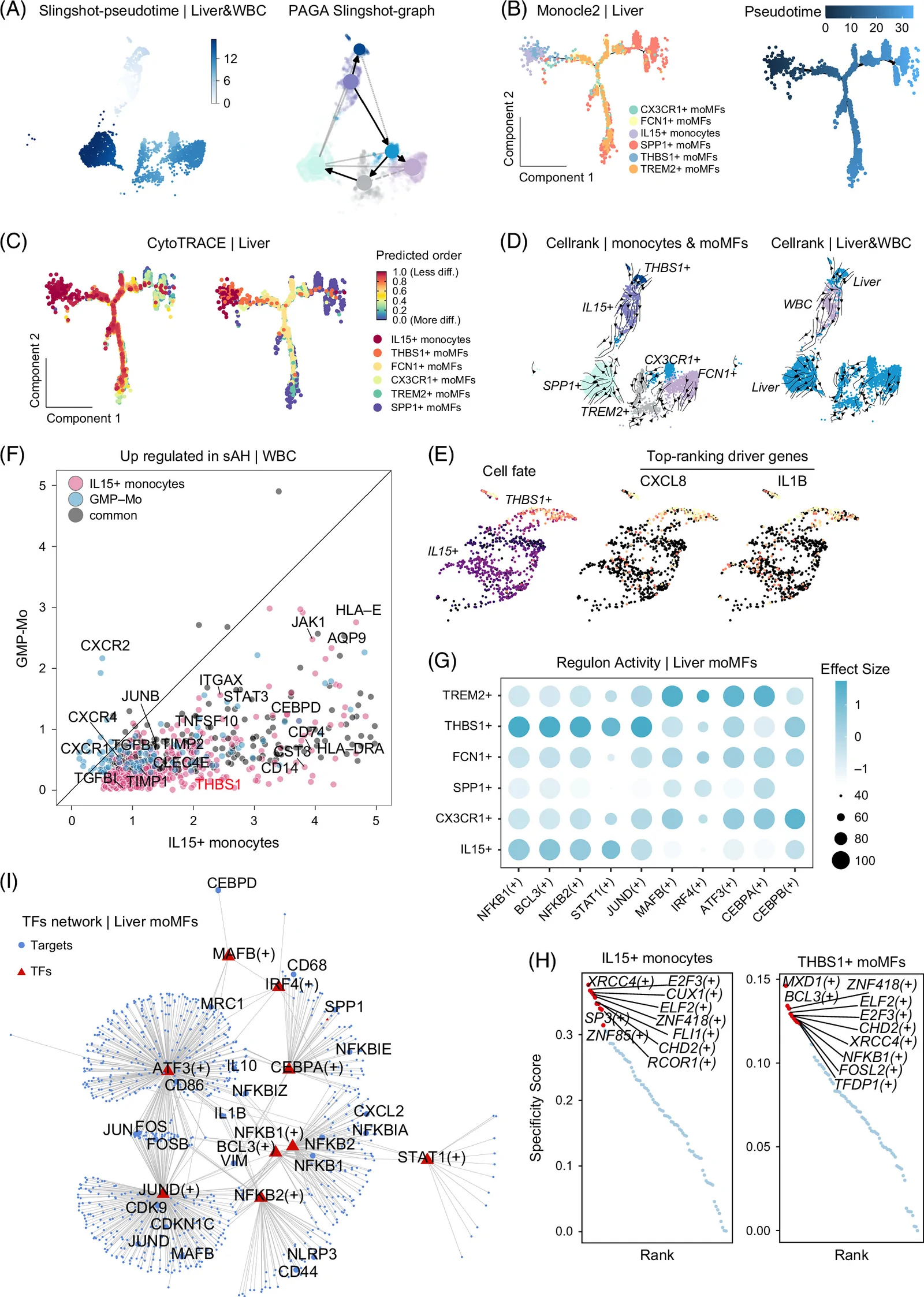
IL15^+^ monocytes differentiate into THBS1^+^ moMFs during ALD progression. (A) Slingshot analysis shows the global trajectory of monocytes and macrophages in the liver and WBC. (B) Monocle2 plots show lineage progression of monocyte and macrophage subsets within the liver. (C) CytoTRACE plots show the differentiation hierarchy of hepatic monocytes and macrophages. (D) CellRank analysis plots show fate probability vectors of liver and WBC on the UMAP projection. (E) Absorption probability plots show differentiation tendencies toward selected cell states and feature plots displaying expression of top‑ranking driver genes. (F) Scatter plots show genes upregulated in circulating monocytes of patients with sAH. (G) Dot plot shows transcription factor motif activity estimated by pySCENIC across hepatic monocyte and macrophage subsets. (H) Plots show the top enriched transcription factor motifs identified in selected monocyte and macrophage subsets. (I) Gene regulatory network plots show transcription factor-target interactions within hepatic monocytes and moMF subsets. Abbreviations: ALD, alcohol-associated liver disease; moMF, monocyte-derived macrophage; sAH, severe alcohol-associated hepatitis; WBC, xxx; UMAP, uniform manifold approximation and projection.

These findings prompted us to further investigate the developmental relationships among disease-associated myeloid subsets. Using multiple pseudotime inference approaches (Slingshot, PAGA, Monocle2, and CytoTRACE), we consistently observed a trajectory from IL15^+^ monocytes toward THBS1^+^ moMFs (Figures 4A–C). Based on Cellrank analysis, we found that CXCL8 and IL1β may be the key factors driving this differentiation (Figures 4D, E). We next compare genes upregulated in IL15^+^ monocytes and GMP‑Mo in sAH WBC samples. IL15^+^ monocytes showed prominent induction of THBS1 together with key inflammatory and macrophage‑priming genes (ITGAX, STAT3, JUNB, TNFSF10, and CEBPB), indicating that they are transcriptionally poised to differentiate into THBS1^+^ moMFs. In contrast, GMP‑Mo upregulated progenitor‑ and chemotaxis‑related genes such as CXCR2, CXCR4, AQP9, and JAK1, consistent with a more immature, precursor‑like state (Figure 4F). Furthermore, the regulatory activities of transcription factors in monocytes and macrophages were systematically evaluated. THBS1^+^ moMFs exhibited markedly increased activity of multiple inflammation-related and macrophage maturation regulons, including NFKB1(+), NFKB2(+), STAT1(+), JUNB (+), and ATF3(+). In contrast, IL15^+^ monocytes showed substantially weaker activation of these regulons (Figure 4G). This progressive enhancement of regulon activity indicates that coordinated transcription factor activation plays a pivotal role in promoting the differentiation and functional remodeling from IL15^+^ monocytes to THBS1^+^ moMFs. In addition, transcription factor regulatory network analysis identified distinct sets of top regulons specific to IL15^+^ monocytes and THBS1^+^ moMFs (Figures 4H, I), further supporting a developmental connection between these 2 subsets.

**FIGURE 5:**
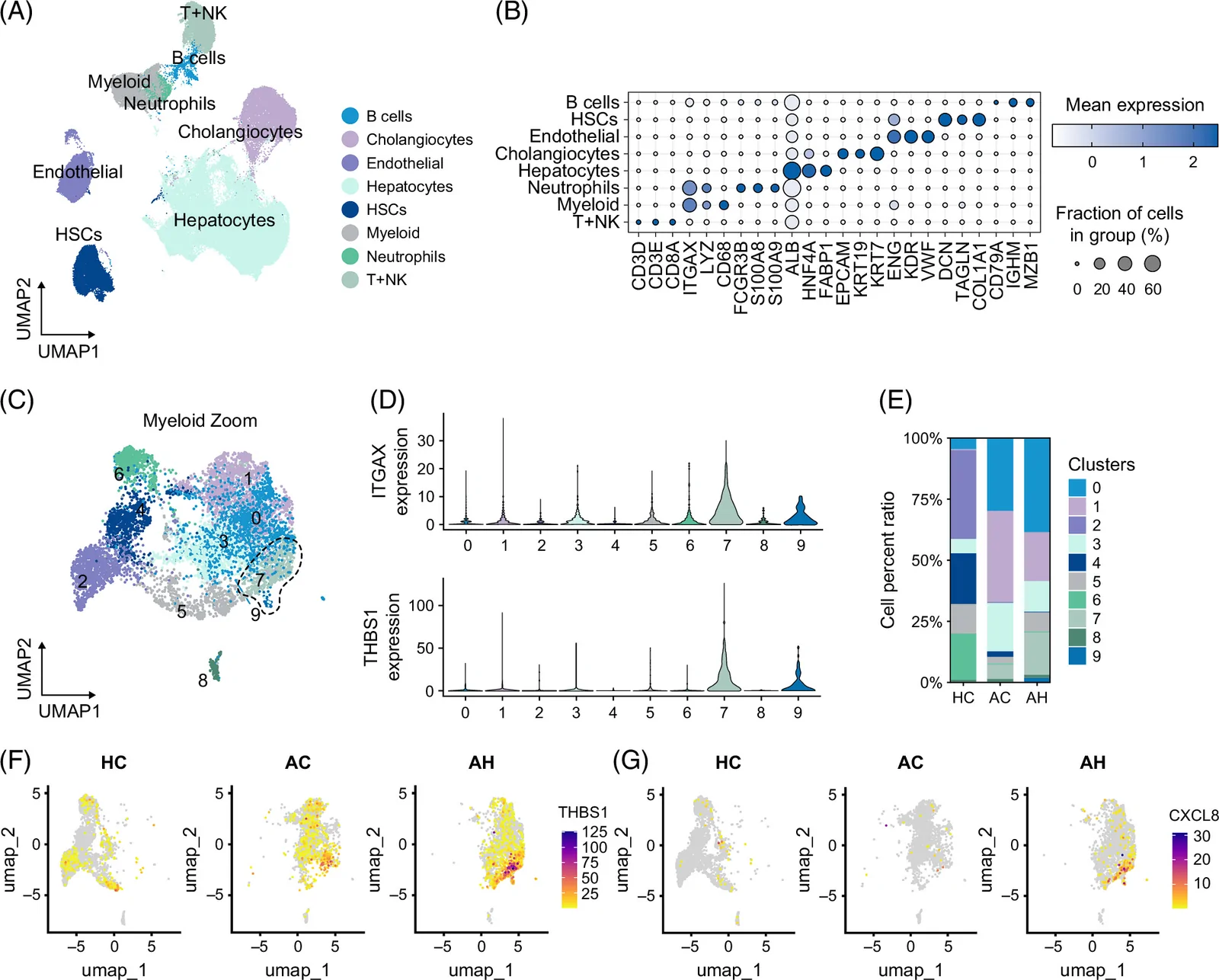
Single-cell profiling identifies distinct hepatic cell populations and stage‑dependent myeloid remodeling in ALD. (A) UMAP plots of hepatic single-cell profiles showing major cell types. (B) Dot plot shows the key marker genes used to define cluster identity and associate each cluster with its corresponding hepatic cell type. (C) UMAP plots highlight the myeloid compartment. (D) Ridge plots show the expression distribution of selected genes across the myeloid clusters. (E) Bar plots show the proportion of myeloid clusters across different disease groups. (F) UMAP feature plots show the spatial distribution of THBS1 expression across samples from different disease states. (G) UMAP feature plots show the spatial distribution of CXCL8 expression across samples from different disease states. Abbreviations: ALD, alcohol-associated liver disease; UMAP, uniform manifold approximation and projection.

In another snRNA‑seq data set, we further validated the expansion and transcriptional features of THBS1^+^ moMFs in ALD. Unsupervised clustering and Uniform Manifold Approximation and Projection visualization similarly identified the major hepatic cell types (Figures 5A, B), and a focused analysis of the myeloid compartment revealed multiple distinct monocyte and macrophage subsets (Figure 5C). Consistent with our previous findings, the proportions of ITGAX^+^/THBS1^+^ moMFs increased progressively from healthy controls to alcoholic cirrhosis and were highest in alcoholic hepatitis (Figures 5D, E). Notably, along this myeloid differentiation trajectory, CXCL8 expression rose in parallel with THBS1 and displayed a tightly correlated pattern, suggesting that CXCL8 acts as a key driver promoting the emergence of THBS1^+^ moMFs (Figures 5F, G).

Together, these findings demonstrate that IL15^+^ monocytes represent an upstream precursor population that progressively differentiates into highly activated THBS1^+^ moMFs during ALD progression. This transition is orchestrated by coordinated transcriptional priming, activation of the inflammatory regulon, and cytokine‑mediated cues such as CXCL8 and IL1β.

### Myeloid remodeling and IL15^+^ monocyte to THBS1^+^ moMF differentiation in MetALD mice

**FIGURE 6:**
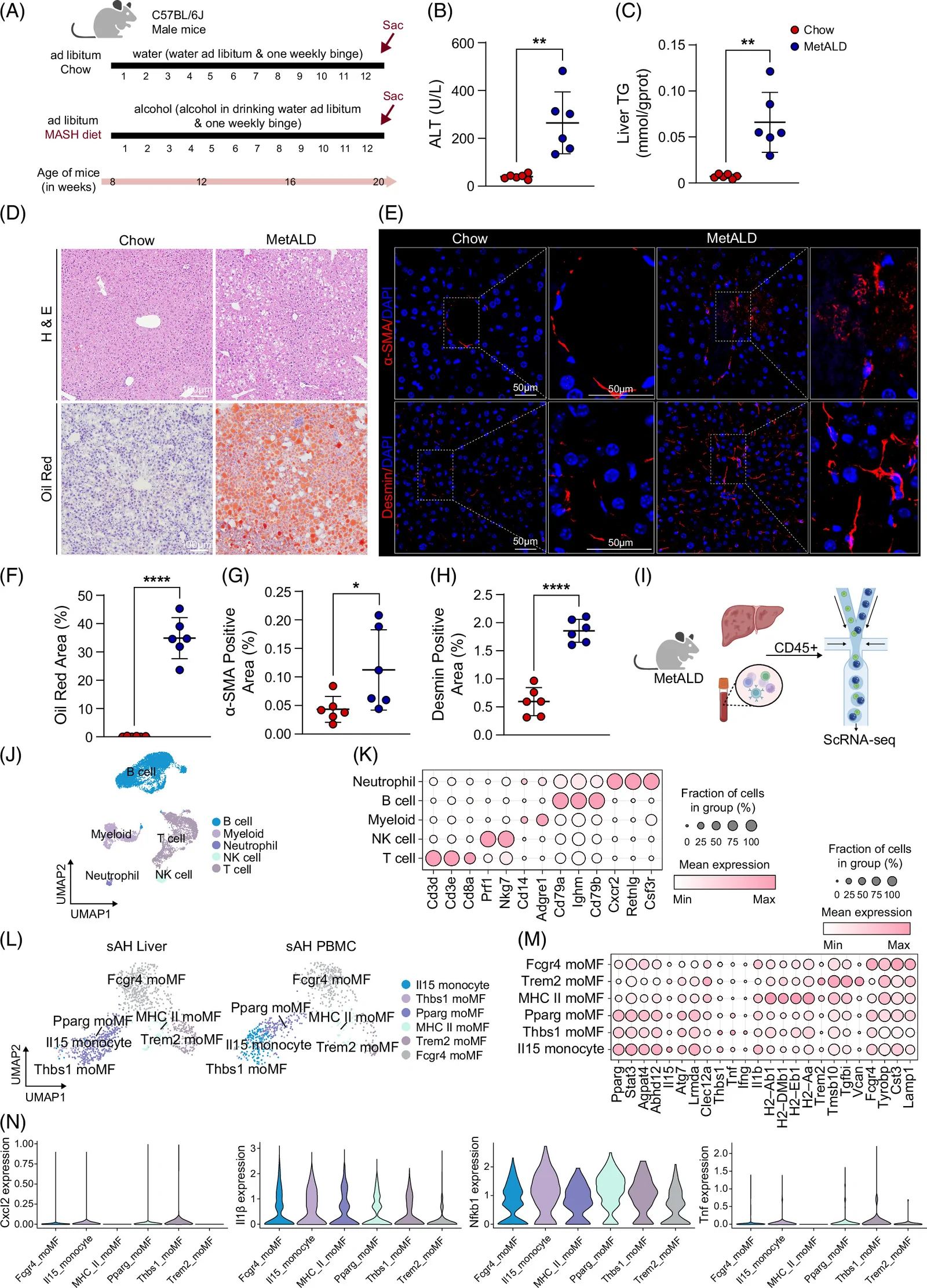
Establishment of the MetALD mouse model and construction of the single-cell immune atlas. (A) Schematic of the MetALD mouse model. (B) Serum ALT levels in chow and MetALD mice. (C) Hepatic TG content in chow and MetALD mice. (D) Representative H&E and Oil Red O staining of liver sections from chow and MetALD mice. (E) Representative α-SMA and desmin immunostaining of liver sections from chow and MetALD mice. (F) Quantification of Oil Red O–positive area%. (G) Quantification of α-SMA positive area%. (H) Quantification of desmin-positive area%. (I) Schematic of scRNA-seq in the MetALD model. (J) UMAP plot shows major immune cell populations distinguished by color. (K) Dot plot shows marker genes used to annotate major immune cell populations. (L) UMAP plots of reclustered myeloid cells from liver and PBMCs show distinct monocyte and macrophage subsets. (M) Dot plot shows marker genes used to annotate myeloid cell subsets. (N) Violin plots show the expression of selected inflammatory genes (Cxcl2, Il1b, and Tnf) and Nfkb1 across myeloid cell subsets identified in the MetALD mouse model. *p<0.05, **p<0.01, ***p<0.001; ns, not significant. Abbreviations: ALD, alcohol-associated liver disease; H&E, hematoxylin and eosin staining; MetALD, metabolic dysfunction–associated steatotic liver disease and alcohol-associated liver disease; PBMC, peripheral blood mononuclear cells; scRNA-seq, single-cell RNA sequencing; TG, triglyceride; UMAP, uniform manifold approximation and projection.

Mice were fed a metabolic dysfunction–associated steatohepatitis–inducing diet and exposed to ethanol under controlled conditions to induce combined metabolic and alcohol-associated liver injury. In accordance with the updated nomenclature, we term this a murine MetALD-like model.^19^,^20^ Based on prior reports,^19^ we established a MetALD mouse model (Figure 6A). Typical features of hepatic injury were induced by this regimen, as evidenced by elevated serum ALT and triglyceride levels, pronounced hepatocellular steatosis on hematoxylin and eosin stainingand Oil Red O staining, and increased expression of HSC markers revealed by alpha-smooth muscle actin and desmin immunostaining, confirming successful model establishment (Figures 6B–H).

Following the validation of the model, CD45^+^ immune cells were isolated from peripheral blood and liver tissues and subjected to scRNA-seq (Figure 6I). Uniform Manifold Approximation and Projection visualization revealed the major immune cell compartments, including B cells, T cells, NK cells, neutrophils, and myeloid cells (Figure 6J), and marker expression was confirmed by dot plot analysis (Figure 6K). High-resolution reclustering of the myeloid compartment identified multiple monocyte and macrophage subsets in both liver and peripheral blood mononuclear cells, including Il15^+^ monocytes, Thbs1^+^ moMFs, Trem2^+^ moMFs, Pparg moMFs, MHC II moMFs, and Fcrg4^+^ moMFs (Figure 6L). The transcriptional signatures of these subsets were clearly demonstrated in the dot plot (Figure 6M) and showed strong concordance with the myeloid populations defined in human ALD. We visualized the expression of key inflammatory genes and an nuclear factor-kappa B–related gene across myeloid clusters in MetALD mice, revealing higher expression in Il15^+^ monocytes and Thbs1^+^ moMFs, consistent with a proinflammatory in vivo transcriptional program (Figure 6N).

**FIGURE 7:**
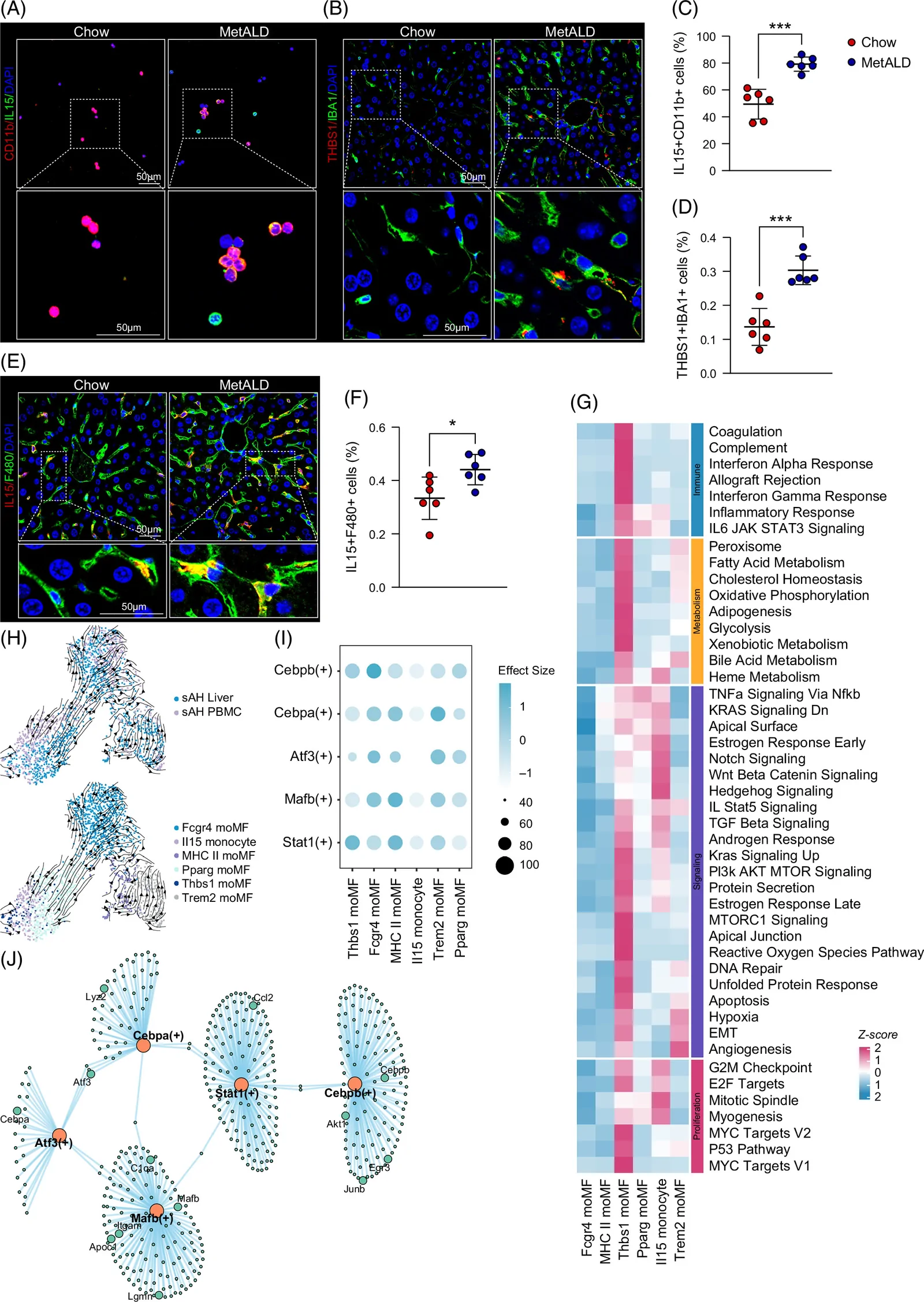
Single-cell landscape of IL15^+^ monocytes and THBS1^+^ macrophages in MetALD. (A) Representative immunofluorescence images of IL15^+^CD11b^+^monocytes in peripheral blood from Chow and MetALD mice. (B) Representative immunofluorescence images of THBS1^+^IBA1^+^ macrophages in liver sections from Chow and MetALD mice. (C) Quantification of IL15^+^CD11b^+^ monocytes in peripheral blood from Chow and MetALD mice. (D) Quantification of THBS1^+^IBA1^+^ macrophages in liver sections from Chow and MetALD mice. (E) Representative immunofluorescence images of IL15^+^F4/80^+^ macrophages in liver sections from Chow and MetALD mice. (F) Quantification of IL15^+^F4/80^+^ cells (%) in liver sections from Chow and MetALD mice. (G) Gene set enrichment analysis plots illustrate pathway enrichment patterns across the identified myeloid cell subsets. (H) Trajectory inference plots depict the inferred developmental relationships among the myeloid cell subsets. (I) Transcription factor activity analysis plots display transcription factor activity patterns across the myeloid cell subsets. (J) Regulatory network reconstruction plots illustrate transcription factor-target gene interactions. *p<0.05, **p<0.01, ***p<0.001; ns, not significant. Abbreviation: MetALD, metabolic dysfunction–associated steatotic liver disease and alcohol-associated liver disease.

Histological analyses showed that Il15^+^ monocytes were markedly increased in the peripheral blood of MetALD mice, while Thbs1^+^ macrophages were prominently accumulated in the liver compared with controls (Figures 7A–D). Immunofluorescence staining of liver sections further validated a significant increase in IL15^+^F4/80^+^ monocyte/macrophage populations in MetALD mice relative to controls (Figures 7E, F). Gene set enrichment analyses demonstrated that both Il15^+^ monocytes and Thbs1^+^ moMFs were enriched for immune‑related programs, while Thbs1^+^ moMFs additionally exhibited metabolic signatures including lipid metabolism, cholesterol homeostasis, and oxidative phosphorylation (Figure 7G). Trajectory inference further delineated a clear developmental continuum from circulating Il15^+^ monocytes to mature Thbs1^+^ moMFs within the liver (Figure 7H), consistent with the differentiation axis observed in human ALD. Integrating transcription factor activity profiling and regulatory network reconstruction (Figures 7I, J) revealed that inflammation‑related and myeloid‑lineage regulons such as Nfκb1(+), Nfκb2(+), Stat1(+), Mafb(+), and Cebpb(+) exhibited low activity in Il15^+^ monocytes but were strongly activated in Thbs1^+^ moMFs. Their coordinated activation along the differentiation trajectory indicates that these regulons form a core regulatory module driving the transition toward the Thbs1^+^ moMF state.

**FIGURE 8:**
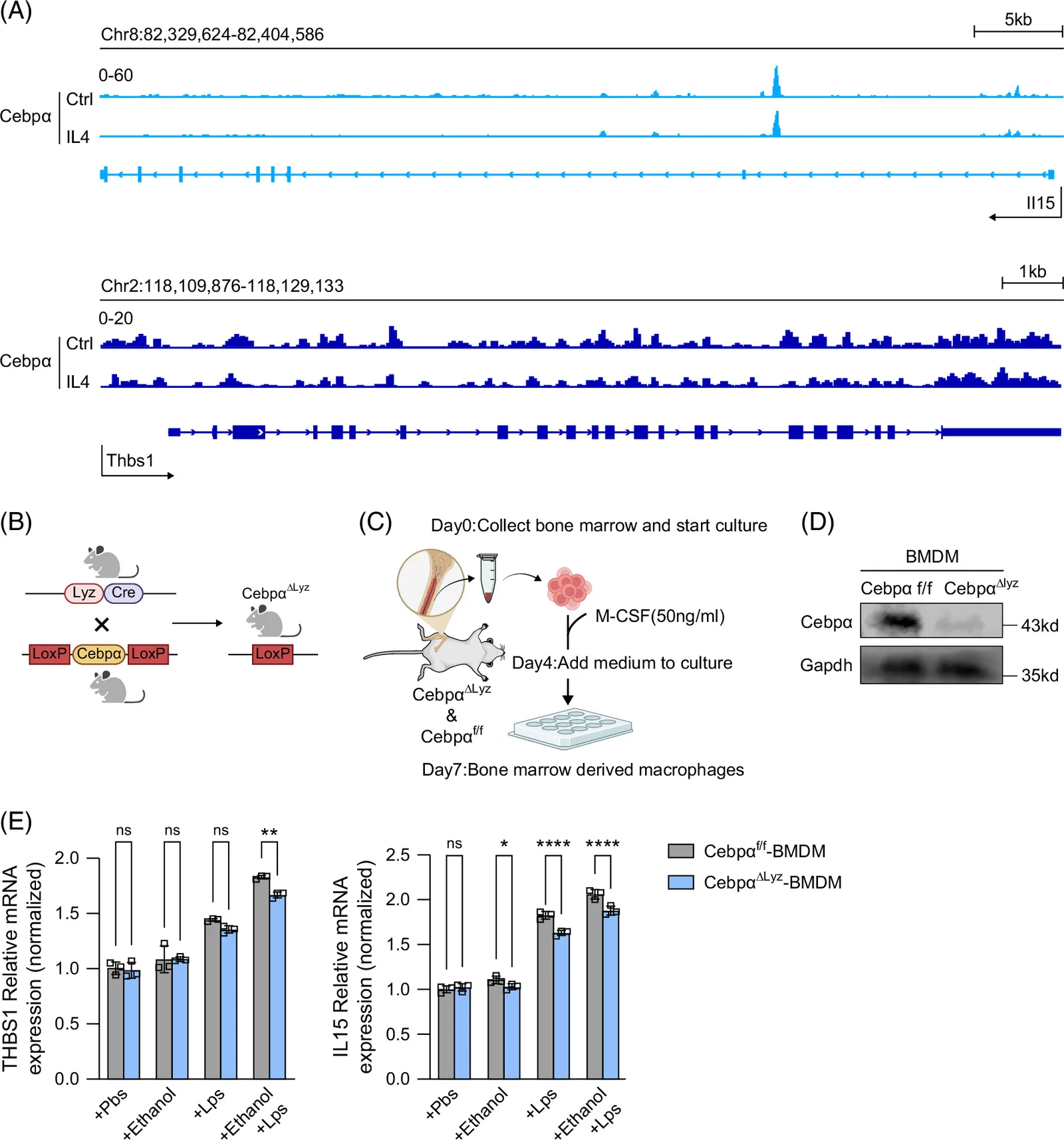
C/EBPα binding at the Il15 and Thbs1 loci and functional requirement for C/EBPα in inflammatory induction. (A) Distribution of Cebpα ChIP‑sequencing tag densities at the Il15 (Chr8) and Thbs1 (Chr2) gene loci under Ctrl and IL‑4 treatment conditions. Gene structures are shown below, with arrows indicating the direction of transcription. Scale bars are indicated as shown. (B) Breeding strategy to generate myeloid-specific Cebpα conditional knockout mice (Cebpα^ΔLyz^). (C) Schematic of BMDM differentiation with M-CSF (50 ng/mL) from Cebpα^f/f^ and Cebpα^ΔLyz^ mice. (D) Immunoblot confirming efficient loss of C/EBPα protein in Cebpα^ΔLyz^ BMDMs. (E) RT-qPCR analysis of Thbs1 and Il15 mRNA expression in Cebpα^f/f^ and Cebpα^ΔLyz^ BMDMs treated with PBS, ethanol, LPS, or ethanol+LPS. *p<0.05, **p<0.01, ***p<0.001; ns, not significant. Abbreviations: BMDM, bone marrow-derived macrophages; LPS, lipopolysaccharide; RT-qPCR, reverse transcription quantitative polymerase chain reaction.

To further evaluate the pySCENIC‑inferred activation of C/EBPα along the IL15^+^ monocyte-to-THBS1^+^ migratory macrophage trajectory, we analyzed publicly available C/EBPα ChIP‑seq data from bone marrow-derived macrophages (BMDMs). Enrichment of C/EBPα was observed at regulatory regions near Il15 and Thbs1, with reduced signal intensity upon IL‑4 stimulation, consistent with polarization‑associated changes in C/EBPα binding (Figure 8A). To functionally validate this regulation, we generated myeloid-specific Cebpα conditional knockout mice (Cebpα^ΔLyz^) and derived BMDMs (Figures 8B, C). Efficient deletion of C/EBPα in BMDMs was confirmed (Figure 8D). Notably, ethanol and lipopolysaccharide (LPS) stimulation induced robust Il15 and Thbs1 expression in control BMDMs, whereas this induction was significantly blunted in Cebpα^ΔLyz^ BMDMs (Figure 8E), supporting a functional requirement for C/EBPα in driving this inflammatory transcriptional program.

### In vitro alcohol‑induced inflammation identifies IL15 and THBS1 as proinflammatory factors

**FIGURE 9:**
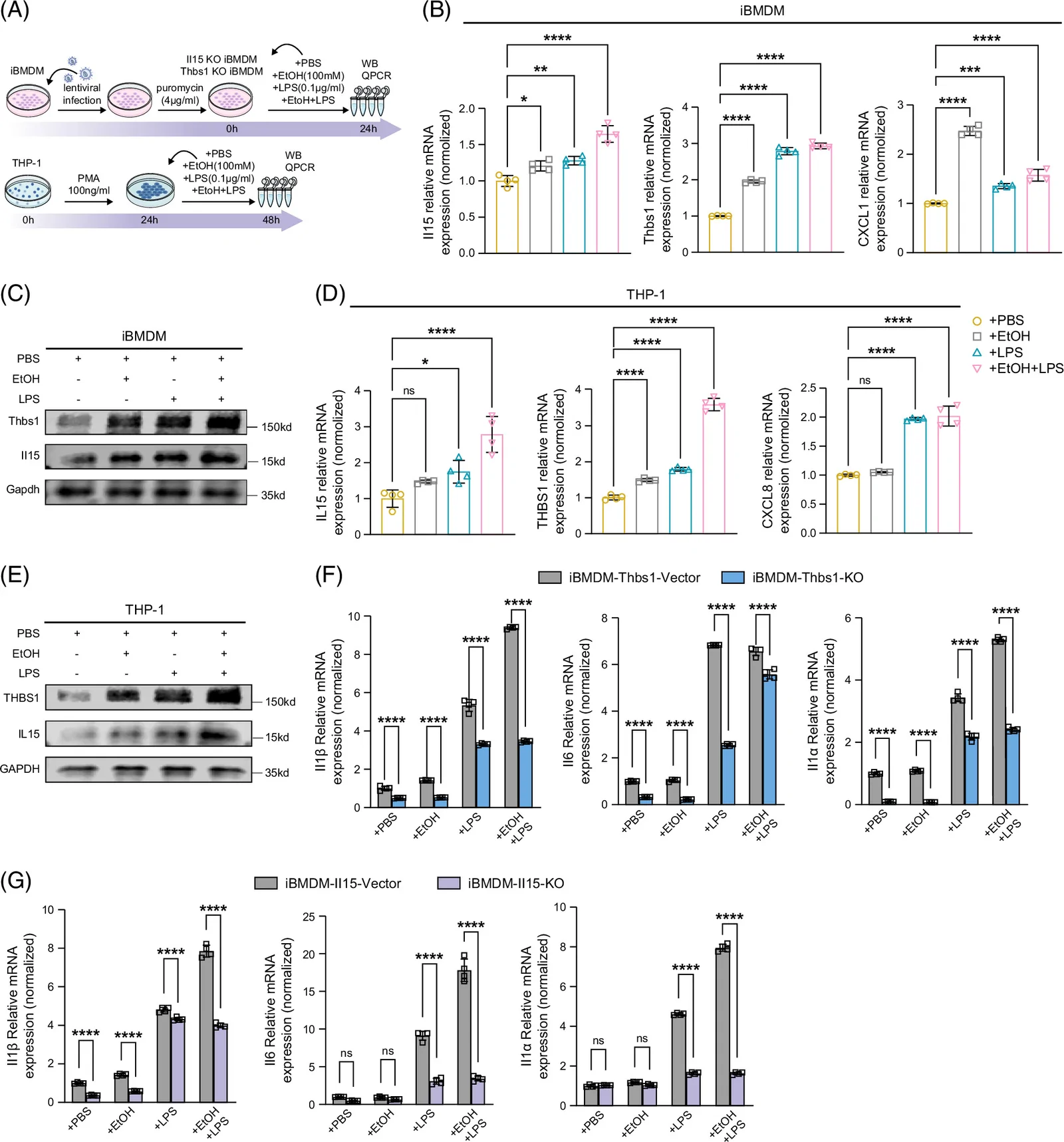
In vitro alcohol‑induced inflammation identifies IL15 and THBS1 as proinflammatory factors. (A) Schematic diagram of ethanol and LPS stimulation in THP-1 and iBMDM cells, and the generation of IL15-KO and THBS1-KO iBMDM cell lines. (B) mRNA expression levels of IL15, THBS1, and CXCL1 in iBMDM cells under different stimulation conditions. (C) Western blot analysis of THBS1 and IL15 expression in iBMDM cells after stimulation. (D) mRNA expression levels of IL15, THBS1, and CXCL8 in THP-1 cells under different stimulation conditions. (E) Western blot analysis of THBS1 and IL15 expression in THP-1 cells after stimulation. (F) mRNA expression levels of IL6, IL1β, IL1α in THBS1-KO iBMDM and THBS1 vector iBMDM cells. (G) mRNA expression levels of IL6, IL1β, IL1α in IL15-KO iBMDM and IL15 vector iBMDM cells. *p<0.05, **p<0.01, ***p<0.001; ns, not significant. Abbreviations: iBMDM, immortalized bone marrow-derived macrophages; LPS, lipopolysaccharide.

Previous studies have reported that ethanol exposure increases the sensitivity of hepatic macrophages to gut-derived LPS transported via the portal circulation, thereby contributing to steatosis, inflammation, and fibrosis in ALD.^21^ In addition, the continuous recruitment of monocyte‑derived infiltrating macrophages expands the hepatic macrophage pool and represents a key contributor to inflammatory progression in ALD.^21^ Based on these observations, we stimulated macrophage cell lines with ethanol and LPS to mimic the inflammatory microenvironment of ALD in vitro (Figure 9A).^22^

To model alcohol-associated inflammatory activation in vitro, we first stimulated murine immortalized bone marrow-derived macrophages macrophages with ethanol and LPS. Combined treatment robustly increased the mRNA and protein expression of Il15 and thbs1, accompanied by a marked induction of Cxcl1, the murine homolog of Cxcl8, whereas either stimulus alone elicited only minimal changes (Figures 9B, C, Supplemental Figures S3A, B, https://links.lww.com/HC9/C476). A similar response pattern was observed in human THP‑1. Ethanol together with LPS significantly enhanced the expression of IL15, THBS1, and CXCL8, with a stronger induction compared with single‑agent stimulation (Figures 9D, E, Supplemental Figure S3C, D, https://links.lww.com/HC9/C476). Taken together, these findings demonstrate that alcohol‑associated inflammatory stimulation elicits a conserved proinflammatory response in macrophages across species, characterized by the coordinated induction of IL15, THBS1, and CXCL8 or CXCL1. This coordinated upregulation supports their roles as inflammation-associated mediators in alcohol-driven immune activation.

After establishing that IL15 and THBS1 are inducible under alcohol-associated inflammatory stimulation, we next examined their functional roles in macrophage inflammatory regulation. IL15‑KO and THBS1‑KO immortalized bone marrow-derived macrophages lines were generated using lentiviral transduction, and efficient gene deletion was verified by qPCR and western blotting (Supplemental Figures S3E–H, https://links.lww.com/HC9/C476). Notably, THBS1 deficiency markedly attenuated the expression of multiple proinflammatory cytokines, both under basal conditions and following ethanol or LPS stimulation, indicating a broad impairment of macrophage inflammatory activation (Figure 9F). IL15‑KO cells exhibited a similarly blunted response, with significant reductions in IL1β, IL6, and IL1α expression under stimulation conditions (Figure 9G). Together, these results establish IL15 and THBS1 as essential regulators of macrophage inflammatory activation in the context of alcohol-associated immune stress.

## DISCUSSION

SAH is characterized by profound and uncontrolled hepatic inflammation, making inflammatory pathways a central focus of clinical research and therapeutic development. moMFs play a pivotal regulatory role in this process. Recent studies have identified multiple functionally distinct macrophage subsets in the livers of patients with sAH and alcohol-associated cirrhosis, each contributing differently to disease progression. However, the differentiation trajectory from monocytes to macrophages and its dynamic regulation remain insufficiently characterized. Therefore, we employed a MetALD mouse model in this study. Although this model has certain limitations, it reproduces part of the pathological features observed in clinical sAH, including marked hepatic inflammation and fibrosis. In this study, by analyzing human and mouse scRNA or snRNA-seq data, we revealed a differentiation trajectory from IL15^+^ monocytes to THBS1^+^ moMFs. This transition represents a key inflammatory pathway in ALD and may offer new opportunities for therapeutic intervention.

Recent studies have highlighted the substantial heterogeneity of hepatic macrophages in advanced ALD. Wang et al systematically characterized macrophages in patients with sAH and alcohol-associated cirrhosis, demonstrating that tissue‑resident KCs are largely replaced by multiple subsets of infiltrating moMFs, including C1Q^+^, S100A8^+^, APOE^+^, TNF^+^, and VSIG4^+^ populations.^11^ Among these, C1Q^+^ macrophages represent an important transitional subset with both proinflammatory and anti‑inflammatory features, underscoring the complexity of macrophage remodeling in sAH. Building upon these findings, we further identified key cellular populations, namely circulating IL15^+^ monocytes and hepatic THBS1^+^ moMFs.

THBS1 has been shown to play an important pathological role in liver diseases. In liver fibrosis, expanded THBS1^+^ macrophages interact with HSCs to drive their activation and profibrotic programs.^23^ Recent single-cell and spatial transcriptomic studies have further expanded the functional spectrum of THBS1, demonstrating that THBS1 promotes profibrotic macrophage subsets and is enriched in fibrotic regions in the kidney and other tissues.^24^ In HBV-related acute-on-chronic liver failure, THBS1 is strongly upregulated, drives systemic inflammation and hepatocyte apoptosis, and correlates with disease severity.^25^ In addition, circulating THBS1 has been reported as a biomarker associated with hepatic lipid accumulation in NAFLD, and myeloid-specific THBS1 deficiency has been shown to confer protection against NAFLD.^26^,^27^ Consistent with these observations, our study identifies THBS1^+^ moMFs as a distinct inflammatory myeloid population that serves as a major source of THBS1 in ALD and plays a critical role in amplifying inflammation and driving disease progression. THBS1 is a secreted extracellular matrix-associated protein with favorable therapeutic tractability. In our cohort analyses, IL15 and THBS1 discriminated sAH, supporting their potential as transcriptomic biomarkers for ALD severity stratification.

IL15 is primarily produced by monocytes,^28^ and current mechanistic studies have largely focused on its regulatory roles in T- and NK-cell immune responses. Previous studies have reported that during NASH, myeloid monocytes promote the formation and maturation of NK precursors through transpresentation of IL15.^29^ Consistent with these observations, our study demonstrates that IL15^+^ monocytes constitute a highly inflammatory circulating population in ALD and serve as the precursor pool that differentiates into pathogenic THBS1^+^ moMFs within the liver, thereby amplifying inflammatory cascades and accelerating disease progression.

Previous studies have shown that the differentiation of circulating monocytes into tissue macrophages is regulated by multiple key signaling pathways. In metabolic dysfunction–associated steatohepatitis, the Notch-RBPJ axis has been identified as a central mechanism driving the transition of monocytes into inflammatory macrophages and monocyte‑derived KCs.^15^ In other tissues, such as the lung and intestine, locally derived growth factors and inflammatory cues have likewise been shown to shape monocyte maturation into tissue‑specific macrophage populations.^14^,^30^ Collectively, these findings highlight the strong context‑dependence of monocyte‑to‑macrophage differentiation and its regulation by multiple finely tuned signaling pathways. By integrating human scRNA-seq or snRNA-seq data sets and validating the findings in our established mouse scRNA-seq model, we identified for the first time a differentiation trajectory from IL15^+^ monocytes to THBS1^+^ moMFs. This pathogenic axis not only reveals how peripheral inflammatory monocytes drive the emergence of aberrant macrophage phenotypes within the liver but also provides important mechanistic insights into macrophage remodeling in ALD. Previous studies have demonstrated that CXCL8 not only participates in the recruitment of monocytes and macrophages but also modulates their functional states through CXCR1/2‑mediated signaling.^31^ In addition, CXCL8^+^ neutrophils promote the progression of sAH.^16^,^32^ Our data further indicate that CXCL8 serves as a key driver promoting the differentiation of IL15^+^ monocytes into THBS1^+^ moMFs.

In conclusion, our study reveals that distinct monocyte and macrophage subsets form a continuous dynamic destiny from IL15^+^ monocytes to THBS1^+^ moMFs during the progression of ALD. This differentiation process is accompanied by pronounced amplification of inflammatory responses and contributes substantially to the remodeling of the sAH-specific inflammatory microenvironment. The abnormal expansion and strong proinflammatory activity of these 2 populations are closely associated with disease severity and poor clinical outcomes, indicating their potential prognostic value. This work deepens our understanding of inflammation-driven monocyte and macrophage biology and provides new avenues for developing therapeutic strategies for sAH by regulating cell fate.

This study has several limitations. First, although THBS1^+^ moMFs were identified as a key pathogenic population in ALD, their interactions with other hepatic cell types, particularly HSCs, were not systematically analyzed, leaving their role in fibrotic remodeling unresolved. Second, the IL15^+^ to THBS1^+^ moMF differentiation trajectory was inferred solely from scRNA-seq–based computational modeling and lacks in vivo validation using genetic tools such as IL15 knockout or lineage-tracing mice. Third, the relatively small number of scRNA-seq and snRNA-seq samples may limit the resolution of macrophage subclusters and the generalizability of our findings. Future studies with larger cohorts and functional experiments will be necessary to validate and extend these observations.

## Supplementary Material
