# Supplementary material for: THBS1+ macrophages fuel pathogenic inflammation in alcohol-associated liver disease
Source: Hepatol Commun. 2026 Sep 18;10(10):e1046. doi: 10.1097/HC9.0000000000001046 (PMC13596963; doi:10.1097/HC9.0000000000001046)

SFig1. Characterization of myeloid cell subsets and their functional states in ALD

(A) The dot plot displays canonical marker genes used to define and annotate myeloid cell subsets. (B) Circular plot illustrates cytokine response signatures in IL15$^+$ monocytes. (C) Stacked bar plots show the distribution of myeloid subsets across different tissue sources and disease groups. (D) Heatmap show regulon activity scores across myeloid subsets derived from SCENIC analysis. (E) Bar plots show the relative proportions of major myeloid subsets across healthy, AC, and sAH liver samples. (F) Boxplot shows the abundance of CX3CR1$^+$ moMFs across disease groups. (G) Boxplot shows the abundance of FCN1$^+$ moMFs across disease groups. (H) Boxplot shows the abundance of TREM2$^+$ moMFs across disease groups. (I) Boxplot shows the abundance of SPP1$^+$ moMFs across disease groups. (J) Boxplots show IL15 and THBS1 gene expression (log (TPM+1)) in HC, AC, and AH samples from the GSE143318 cohort. Each dot represents an individual sample. *$p < 0.05$, **$p < 0.01$, ***$p < 0.001$; ns, not significant.

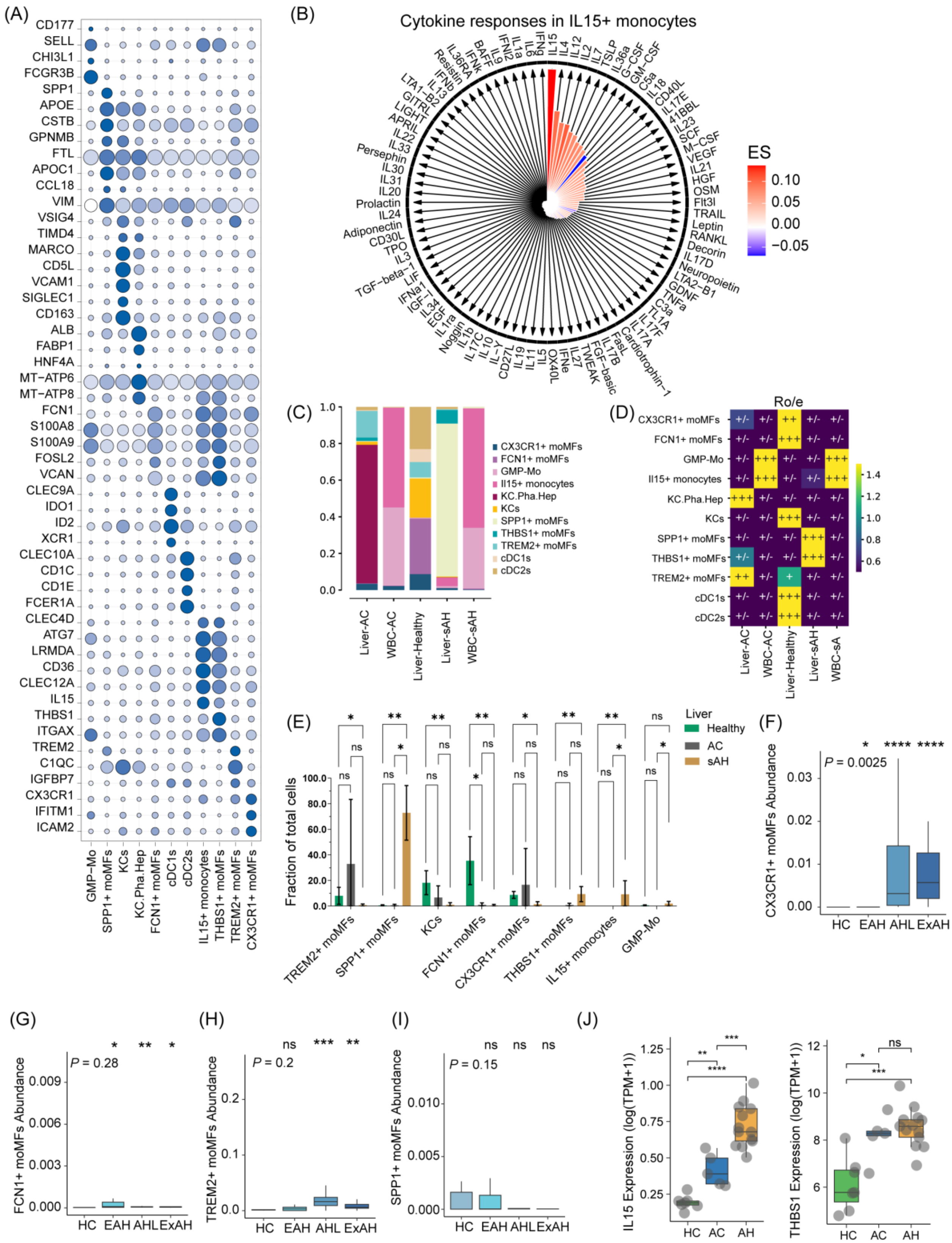

(A)
CD177
SELL
CHI3L1
FCGR3B
SPP1
APOE
CSTB
GPNMB
FTL
APOC1
CCL18
VIM
VSIG4
TIMD4
MARCO
CD5L
VCAM1
SIGLEC1
CD163
ALB
FABP1
HNF4A
MT-ATP6
MT-ATP8
FCN1
S100A8
S100A9
FOSL2
VCAN
CLEC9A
IDO1
ID2
XCR1
CLEC10A
CD1C
CD1E
FCER1A
CLEC4D
ATG7
LRMDA
CD36
CLEC12A
IL15
THBS1
ITGAX
TREM2
C1QC
IGFBP7
CX3CR1
IFITM1
ICAM2
GMP-Mo
SPP1+ moMFs
KCs
KC.Pha.Hep
FCN1+ moMFs
cDC1s
cDC2s
IL15+ monocytes
THBS1+ moMFs
TREM2+ moMFs
CX3CR1+ moMFs
(B)
Cytokine responses in IL15+ monocytes
ES
0.10
0.05
0.00
−0.05
IL15
IL4
IL12
IL2
IL7
TSLP
IL36a
G-CSF
GM-CSF
C5a
IL18
CD40L
IL17E
41BBL
IL23
SCF
M-CSF
VEGF
IL21
HGF
OSM
Flt3l
TRAIL
Leptin
RANKL
Decorin
IL17D
Neuropoietin
LTA2-B1
GDNF
TNFa
C3a
IL1A
IL17F
IL17A
Cardiotrophin-1
IL17B
FasL
FGF-basic
TWEAK
IL27
IFNe
OX40L
IL5
IL11
IL19
CD27L
IL-Y
IL10
IL17C
IL1b
Noggin
IL1ra
EGF
IL34
IGF-1
IFNa1
LIF
TGF-beta-1
IL3
TPO
CD30L
Adiponectin
IL24
Prolactin
IL20
IL31
IL30
Persephin
IL33
IL22
APRIL
LIGHT
GITRL
LTA1-B2
IL13
IFNb
Resistin
IL36RA
IFNk
BAFF
IL9
IFNl2
IL1a
IL6
IFNg
(C)
1.0
0.8
0.6
0.4
0.2
0.0
CX3CR1+ moMFs
FCN1+ moMFs
GMP-Mo
Il15+ monocytes
KC.Pha.Hep
KCs
SPP1+ moMFs
THBS1+ moMFs
TREM2+ moMFs
cDC1s
cDC2s
Liver-AC
WBC-AC
Liver-Healthy
Liver-sAH
WBC-sAH
(D)
Ro/e
CX3CR1+ moMFs
FCN1+ moMFs
GMP-Mo
Il15+ monocytes
KC.Pha.Hep
KCs
SPP1+ moMFs
THBS1+ moMFs
TREM2+ moMFs
cDC1s
cDC2s
Liver-AC
WBC-AC
Liver-Healthy
Liver-sAH
WBC-sA
1.4
1.2
1.0
0.8
0.6
(E)
Liver
Healthy
AC
sAH
Fraction of total cells
100.0
80.0
60.0
40.0
20.0
0.0
TREM2+ moMFs
SPP1+ moMFs
KCs
FCN1+ moMFs
CX3CR1+ moMFs
THBS1+ moMFs
IL15+ monocytes
GMP-Mo
(F)
CX3CR1+ moMFs Abundance
P = 0.0025
0.03
0.02
0.01
0.00
HC
EAH
AHL
ExAH
(G)
FCN1+ moMFs Abundance
P = 0.28
0.009
0.006
0.003
0.000
HC
EAH
AHL
ExAH
(H)
TREM2+ moMFs Abundance
P = 0.2
0.2
0.1
0.0
HC
EAH
AHL
ExAH
(I)
SPP1+ moMFs Abundance
P = 0.15
0.010
0.005
0.000
HC
EAH
AHL
ExAH
(J)
IL15 Expression (log(TPM+1))
1.25
1.00
0.75
0.50
0.25
HC
AC
AH
THBS1 Expression (log(TPM+1))
12
10
8
6
HC
AC
AH

SFig2. Transcriptomic and pathway characteristics of IL15$^+$ monocytes and THBS1$^+$ moMFs

(A) Volcano plot displays differentially expressed genes (DEGs) in IL15$^+$ monocytes. (B) KEGG and GO pathway enrichment analyses of IL15$^+$ monocytes reveal major biological processes and signaling pathways. (C) Volcano plot displays DEGs in THBS1$^+$ moMFs. (D) KEGG and GO pathway enrichment analyses of THBS1$^+$ moMFs reveal major biological processes and signaling pathways. (E) Violin plots show inflammation-related signature scores across myeloid subsets. (F) Violin plots show ECM-regulator signature scores across myeloid subsets. (G) Violin plots show collagen fibril organization signature scores across myeloid subsets. *$p < 0.05$, **$p < 0.01$, ***$p < 0.001$; ns, not significant.

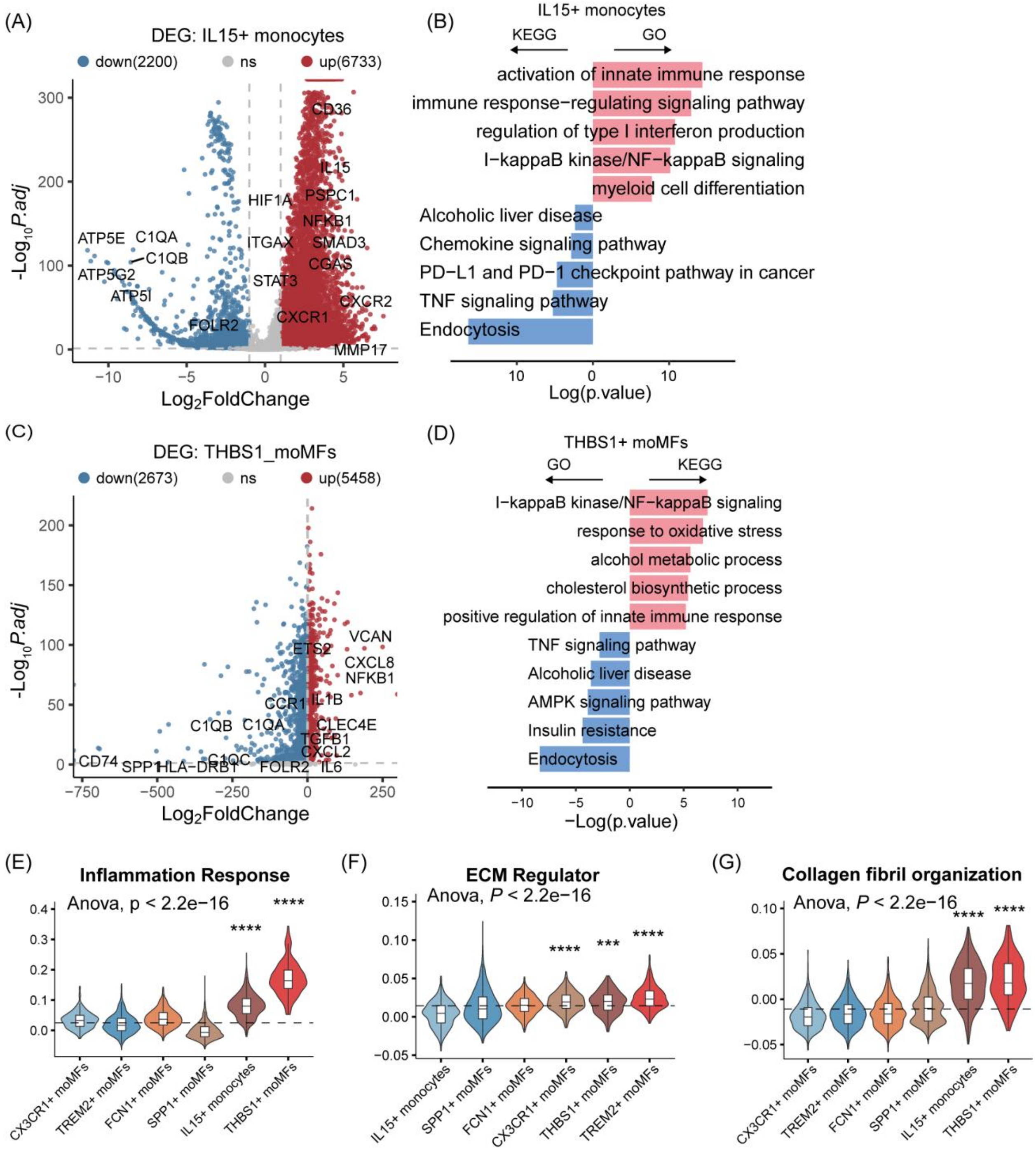

(A)
DEG: IL15+ monocytes
down(2200)
ns
up(6733)
CD36
IL15
HIF1A
PSPC1
NFKB1
ATP5E
C1QA
ITGAX
SMAD3
C1QB
CGAS
ATP5G2
STAT3
ATP5I
CXCR2
FOLR2
CXCR1
MMP17
-Log10P.adj
Log2FoldChange
(B)
IL15+ monocytes
KEGG
GO
activation of innate immune response
immune response−regulating signaling pathway
regulation of type I interferon production
I−kappaB kinase/NF−kappaB signaling
myeloid cell differentiation
Alcoholic liver disease
Chemokine signaling pathway
PD−L1 and PD−1 checkpoint pathway in cancer
TNF signaling pathway
Endocytosis
Log(p.value)
(C)
DEG: THBS1_moMFs
down(2673)
ns
up(5458)
VCAN
ETS2
CXCL8
NFKB1
CCR1
IL1B
C1QB
C1QA
CLEC4E
TGFB1
CXCL2
C1QC
CD74
SPP1
HLA−DRB1
FOLR2
IL6
-Log10P.adj
Log2FoldChange
(D)
THBS1+ moMFs
GO
KEGG
I−kappaB kinase/NF−kappaB signaling
response to oxidative stress
alcohol metabolic process
cholesterol biosynthetic process
positive regulation of innate immune response
TNF signaling pathway
Alcoholic liver disease
AMPK signaling pathway
Insulin resistance
Endocytosis
−Log(p.value)
(E)
Inflammation Response
Anova, p < 2.2e−16
CX3CR1+ moMFs
TREM2+ moMFs
FCN1+ moMFs
SPP1+ moMFs
IL15+ monocytes
THBS1+ moMFs
(F)
ECM Regulator
Anova, P < 2.2e−16
IL15+ monocytes
SPP1+ moMFs
FCN1+ moMFs
CX3CR1+ moMFs
THBS1+ moMFs
TREM2+ moMFs
(G)
Collagen fibril organization
Anova, P < 2.2e−16
CX3CR1+ moMFs
TREM2+ moMFs
FCN1+ moMFs
SPP1+ moMFs
IL15+ monocytes
THBS1+ moMFs

SFig3. IL15 and THBS1 expression and knockout validation

(A) Quantification of THBS1 protein levels in iBMDM cells. (B) Quantification of IL15 protein levels in iBMDM cells. (C) Quantification of THBS1 protein levels in THP-1 cells. (D) Quantification of IL15 protein levels in THP-1 cells. (E) Western blot analysis confirming successful knockout of THBS1 in iBMDM cells. (F) qPCR analysis measuring THBS1 mRNA levels in iBMDM cells transduced with vector control or THBS1-KO constructs. (G) Western blot validation of IL15 knockout in iBMDM cells. (H) qPCR analysis measuring IL15 mRNA levels in iBMDM cells transduced with vector control or IL15-KO constructs. *$p < 0.05$, **$p < 0.01$, ***$p < 0.001$; ns, not significant.

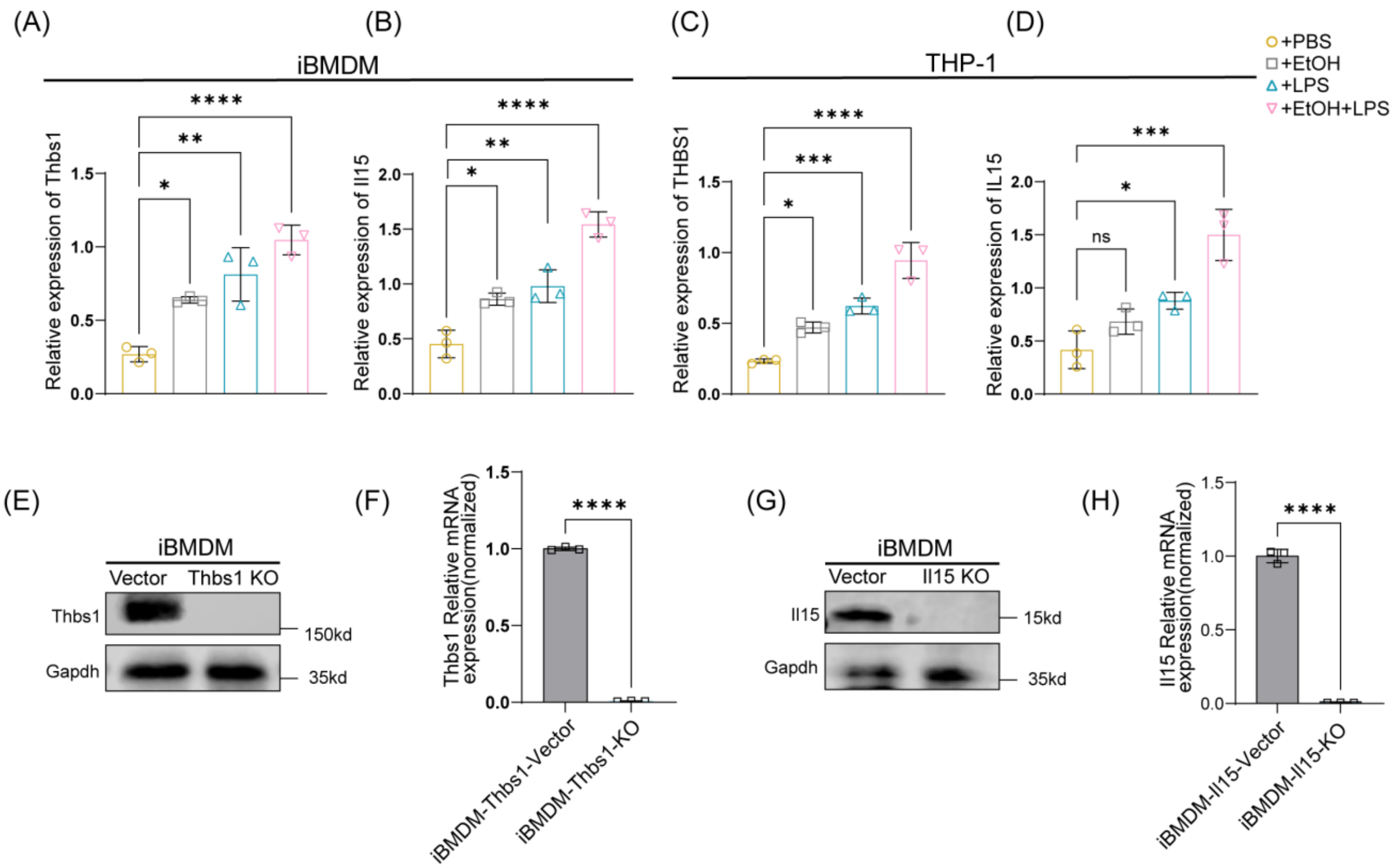

Supplement: Supplementary file 1 [file hc9-10-e1046-s002.pdf]
