# Supplementary material for: THBS1+ macrophages fuel pathogenic inflammation in alcohol-associated liver disease
Source: Hepatol Commun. 2026 Sep 18;10(10):e1046. doi: 10.1097/HC9.0000000000001046 (PMC13596963; doi:10.1097/HC9.0000000000001046)
Supplement: Supplementary file 2 [file hc9-10-e1046-s003.docx]

**Supplementary Methods**

**Human peripheral blood collection**

Human peripheral blood samples were obtained from the First Affiliated Hospital of Anhui Medical University. The study was conducted in accordance with the Declarations of Helsinki and Istanbul and was reviewed and approved by the Institutional Research Ethics Committee of the First Affiliated Hospital of Anhui Medical University (approval number 2022173). Written informed consent was obtained from all participants. Clinical information is provided in Supplementary Table 1.

**Animals**

Male wild‑type C57BL/6J mice (7 weeks old) were obtained from GemPharmatech (Nanjing, China). Mice were maintained under specific pathogen‑free conditions with a standard 12hour light/12hour dark cycle. Modeling was performed after one week of environmental acclimation. All animal procedures were conducted in accordance with the ethical guidelines for the care and use of laboratory animals. All procedures were approved by the Institutional Animal Care and Use Committee (IACUC) of Anhui Medical University.

**MetALD mice model**

Mice were fed a high fat–cholesterol–sucrose diet (MASH diet; Research Diets, Inc., USA, D09061704) or a standard chow diet ad libitum for 3 months. MASH diet–fed and chow-fed mice were randomly divided into two subgroups to receive either alcohol or water. The alcohol groups received 10% alcohol in drinking water ad libitum and 5 g/kg alcohol gavage weekly, whereas the water groups had access to regular water ad libitum and received a weekly water gavage for 3 months. Mice that received both the MASH diet and alcohol were referred to as MetALD mice. All the mice were euthanized 9 hours after the final alcohol or water gavage. Blood and liver tissues were collected immediately after sacrifice, processed, and stored at -80°C for subsequent analyses. This paradigm was designed to model combined metabolic and alcohol-related liver injury and is referred to here as a MetALD(-like) mouse model.

**Cell lines culture.**

The human monocytic cell line THP‑1 and the mouse immortalized bone marrow–derived macrophage cell line iBMDM were used in this study. Mycoplasma contamination was excluded using PCR‑based methods. THP‑1 cells were cultured in RPMI1640 medium supplemented with 10% FBS and 1% penicillin–streptomycin (P/S). iBMDM cells were maintained in DMEM containing 10% FBS and 1% penicillin–streptomycin (P/S). All cells were incubated at 37°C in a humidified atmosphere with 5% CO₂.

**Serum ALT**

Serum alanine aminotransferase (ALT) level was measured using an automatic biochemical analyzer (Myriad, Shenzhen, China) according to the manufacturer’s instructions.

**Liver tissue triglycerides**

Liver triglyceride (TG) content was quantified in 10% liver tissue homogenates using commercial assay kits (Jiancheng Bioengineering Institute, Nanjing, China) following the manufacturer's protocol.

**Histology**

Liver tissue was fixed, paraffin-embedded, and stained with hematoxylin and eosin (H&E) for injury assessment. Hepatic steatosis was evaluated using Oil Red O on OCT-embedded sections (8 μm).

**Western blotting**

Proteins were extracted from THP-1 or iBMDM cells using RIPA lysis buffer (Beyotime, Shanghai, China) containing protease and phosphatase inhibitors. Proteins probed with the following antibodies: anti-IL15(1:500, Santa, USA), anti-THBS1(1:1000, Proteintech, China). All antibodies used in the experiments are detailed in Supplementary Table 2.

**Quantitative real-time polymerase chain reaction (qRT-PCR)**

Total RNA was extracted using TRIZOL reagent (Takara, Tokyo, Japan). Reverse transcription was performed using the Vazyme RT III Kit (Vazyme, Nanjing, China). Gene expression levels were quantified using SYBR Green reagent (Vazyme, Nanjing, China) on a real-time PCR system (Thermo Fisher Scientific, USA). GAPDH levels were used for normalization. All primer sequences used in this study are provided in Supplementary Table 3.

**Immunofluorescence**

Paraffin‑embedded mouse sections (3.5 μm) were deparaffinized in xylene, rehydrated through graded ethanol, and subjected to antigen retrieval in citrate buffer (pH=6) under high‑pressure conditions. Autofluorescence was quenched using a bleaching solution combined with strong light exposure. Sections were blocked with goat serum at 37°C for 30 minutes, followed by incubation with diluted primary antibodies at 4°C overnight and rewarming at 37°C the next day. After washing, fluorescent secondary antibodies were applied and incubated at 37°C for 30 minutes. Sections were washed, counterstained with DAPI, and mounted with antifade mounting medium. All antibodies used in the experiments are detailed in Supplementary Table 2. Images were acquired using confocal microscope (SUNNY CSIM 130, Suzhou, China).

**Isolation of Liver Non‑Parenchymal Cells (NPCs)**

Mouse liver NPCs were isolated using a modified two‑step enzymatic perfusion procedure. Briefly, the liver was perfused in situ with pre‑warmed D‑Hanks solution containing 1 mM HEPES and 0.5 mM EGTA, followed by a second perfusion with D‑Hanks supplemented with 0.01% Collagenase IV and 0.001% DNase I. After perfusion, liver gently dissociated in cold DMEM and passed through a cell strainer. The suspension was centrifuged at 50 g for 3 min to sediment hepatocytes. The cell suspension was subjected to Percoll density gradient centrifugation to enrich non‑parenchymal cells. After centrifugation, the collected cells were washed and resuspended in PBS containing BSA to obtain purified NPCs.

**Isolation of Mouse Peripheral Blood Mononuclear Cells (PBMCs)**

Peripheral blood was collected from mice using EDTA‑coated tubes via retro‑orbital bleeding. The blood was diluted 1:1 with sterile PBS and carefully layered onto an equal volume of Ficoll, avoiding disturbance of the interface. Samples were centrifuged at 800 g for 25-30 minutes with low acceleration and deceleration to achieve proper density separation. After centrifugation, the mononuclear cell layer located at the plasma-Ficoll interface was gently aspirated and transferred into a new tube. The collected cells were washed with PBS to obtain purified mouse PBMCs for subsequent single‑cell sequencing.

**Single-cell transcriptomic sequencing**

For mouse liver non-parenchymal cells (NPCs) and peripheral blood mononuclear cells (PBMCs), scRNA libraries were generated using the DNBelab C Series High‑throughput Single Cell RNA Library Prep Set (MGI, #940‑000047‑00). Sequencing was performed on the DNBSEQ T7 platform to obtain high‑throughput transcriptomic data. Raw reads were processed with DNBC4Tools for quality control, demultiplexing, and alignment to the mouse reference genome, yielding gene‑barcode count matrices for downstream analysis. Single‑cell transcriptomic profiling was performed on NPCs and PBMCs isolated from a single mouse (n = 1).

**Construction of stable knockout cell lines**

The IL15 and THBS1 knockout plasmids were obtained from Corues Biotechnology (China). Lentiviral packaging was performed by seeding 293T cells in 6well plates to reach approximately 70–80% confluence the following day. Plasmid DNA (PAX 5 µg, PMD 2.5 µg, and transfer vector 5 µg) and Lipo8000 (24 µL) were each mixed with 125 µL Opti‑MEM, incubated at room temperature for 5 minutes to form complexes, and then added to the cells. After 12 hours, the medium was replaced with fresh complete medium. Viral supernatants were collected approximately 48 hours post‑transfection and subsequently filtered. Lentiviral infection was carried out by adding viral supernatant to the cells at a 2:1 ratio of complete medium to virus in 6‑well plates, supplemented with polybrene (4 µg/mL) to enhance infection efficiency. After a 12 hours infection period, the medium was replaced with fresh complete medium. Selection was initiated 48 hours later using puromycin at a final concentration of 4 μg/mL, followed by a second round of infection using the same procedure. Single cell clones were subsequently isolated by limiting dilution and validated. Knockout efficiency was validated by Western blot and quantitative PCR analyses.

**Isolation and differentiation of mouse bone marrow-derived macrophages (BMDMs)**

Femurs and tibias were harvested under sterile conditions and surrounding muscle tissues were removed. Bone marrow cells were flushed from the marrow cavity using a syringe with PBS. The cell suspension was passed through a 70µm cell strainer and collected by centrifugation (300g, 5 min, 4°C). Red blood cells were lysed using RBC lysis buffer for 3 min at room temperature, followed by quenching with excess culture medium and centrifugation.

Cells were resuspended in complete macrophage differentiation medium consisting of DMEM supplemented with 10% fetal bovine serum (FBS), 1% penicillin–streptomycin, and recombinant murine macrophage colony-stimulating factor (M‑CSF, 50 ng/mL). Cells were plated in culture dishes and incubated at 37°C in a humidified atmosphere with 5% CO₂. On day 4, half of the medium was gently replaced with fresh complete medium containing M‑CSF (50 ng/mL). By day 7, adherent cells were considered mature BMDMs.

**Data processing**

The Seurat package was utilized for an in-depth downstream analysis^1^. Cells that exhibited fewer than 200 Unique Molecular Identifiers (UMIs) or had > 20% of their genes originating from mitochondria were excluded from the analysis to ensure data quality. The doublefinder R package was used to identify and discard the presumed doublet^2^. To mitigate batch effects and prevent any undue influence from individual patient characteristics on the analysis, the Harmony algorithm was implemented to effectively remove batch effects within the single-cell data^2^. To facilitate dimensionality reduction and the identification of cell subtypes, the FindVariableFeatures function was employed to select the 3000 most variable genes, which were then scaled appropriately. A principal component analysis (PCA) was subsequently conducted using those variable genes. By utilizing the FindNeighbors function, nearest neighbors for graph clustering were determined based on the top 30 principal components. The FindCluster function was then used to obtain the resulting clusters, and cell visualization was achieved by applying the uniform manifold approximation and projection (UMAP) algorithm. Additionally, gene signatures specific for various cell types were scored within the identified clusters. A differential gene expression analysis between clusters was performed using the "FindAllMarkers" function, with parameters set to min.pct =0.25, logfc.threshold = 0.25, and only.pos = TRUE. The Wilcoxon's rank-sum test, coupled with the Benjamini-Hochberg method, was utilized to determine p-values for the comparisons; thereby ensuring robust statistical significance. To characterize the distribution of cell types, odds ratios were calculated based on established reports to indicate preferential enrichment^3^.

**Gene set enrichment analysis**

Mfuzz package was used for fuzzy clustering analysis of RNA sequencing data to identify genes associated with disease progression^4^. Gene Ontology enrichment analysis was implemented with compareCluster and gseGO functions in clusterProfiler package^5^. Human hallmark gene set was obtained from MSigDB, then gene set variation analysis was implemented with GSVA package. Using AddmoduleScore in Seurat function to evaluate the gene set activity of a single cell. The gene set activity score was measured as the proportion of genes centrally expressed by query genes and their relative expression levels compared with other genes in each cell. Gene sets used in the analyses included “NABA_ECM_REGULATORS’’, ‘‘GOBP_INFLAMMATORY_RESPONSE’’ and “GOBP_COLLAGEN_FIBRIL_ORGANIZATION’’ from MSigDB GO gene sets.

**BayesPrism deconvolution**

BayesPrism deconvolution was applied to cell type deconvolution^6^, and cell specific expression profiles were calculated in two independent data sets, GSE143318^7^ and dbGAP: phs001807.v1.p1^8^, respectively. The default parameters were used in the analysis process.

**Cytokine response and cell polarization analysis**

According to the previous IREA^9^ calculation method of immune dictionary, the cytokine response was evaluated by gene expression matrix.

**Single-cell trajectories**

CytoTRACE, Monocle2 and CellRank were used to infer the developmental trajectory of human mononuclear macrophages^10-12^. CytoTRACE uses log2 to standardize the expression of matrix. The predicted sequence is projected into the neutrophil UMAP space. For Monocle 2, we used the newCellDataSet function to build a new CellDataSet object from the cluster annotated Seurat object. We sort the cells and then use the DDRTree algorithm to reduce the dimension and sort the cells along with the trajectory. In addition, the CytoTRACE score is projected onto the Monocle track. Using CellRank software package, the pseudotime and transcriptome similarity information are combined to calculate the cell-cell transition matrix. Focusing on the classical monocyte-macrophage system, the genes expressed related to the absorption probability towards the terminal state of THBS1⁺ moMFs were identified as potential lineage drivers.

**Regulon network**

The regulatory subnetwork was explored using the software package pySCENIC^13^, which analyzed the co-expression of transcription factors and their presumed target genes. We use default parameters to construct a gene regulatory network and score it. The regulator activity of each cell was calculated as the average normalized expression of the presumed target gene.

References

1.	Macosko EZ, Basu A, Satija R, et al. Highly Parallel Genome-wide Expression Profiling of Individual Cells Using Nanoliter Droplets. *Cell*. 2015;161(5):1202–1214. doi:10.1016/j.cell.2015.05.002

2.	McGinnis CS, Murrow LM, Gartner ZJ. DoubletFinder: Doublet Detection in Single-Cell RNA Sequencing Data Using Artificial Nearest Neighbors. *Cell Syst*. 2019;8(4)doi:10.1016/j.cels.2019.03.003

3.	Xue R, Zhang Q, Cao Q, et al. Liver tumour immune microenvironment subtypes and neutrophil heterogeneity. *Nature*. 2022;612(7938):141–147. doi:10.1038/s41586-022-05400-x

4.	Shen B, Yi X, Sun Y, et al. Proteomic and Metabolomic Characterization of COVID-19 Patient Sera. *Cell*. 2020;182(1)doi:10.1016/j.cell.2020.05.032

5.	Xu S, Hu E, Cai Y, et al. Using clusterProfiler to characterize multiomics data. *Nat Protoc*. 2024;19(11):3292–3320. doi:10.1038/s41596-024-01020-z

6.	Tran KA, Addala V, Johnston RL, et al. Performance of tumour microenvironment deconvolution methods in breast cancer using single-cell simulated bulk mixtures. *Nat Commun*. 2023;14(1):5758. doi:10.1038/s41467-023-41385-5

7.	Hyun J, Sun Z, Ahmadi AR, et al. Epithelial splicing regulatory protein 2-mediated alternative splicing reprograms hepatocytes in severe alcoholic hepatitis. *J Clin Invest*. 2020;130(4):2129–2145. doi:10.1172/JCI132691

8.	Argemi J, Latasa MU, Atkinson SR, et al. Defective HNF4alpha-dependent gene expression as a driver of hepatocellular failure in alcoholic hepatitis. *Nat Commun*. 2019;10(1):3126. doi:10.1038/s41467-019-11004-3

9.	Cui A, Huang T, Li S, et al. Dictionary of immune responses to cytokines at single-cell resolution. *Nature*. 2023;625(7994):377–384. doi:10.1038/s41586-023-06816-9

10.	Gulati GS, Sikandar SS, Wesche DJ, et al. Single-cell transcriptional diversity is a hallmark of developmental potential. *Science*. 2020;367(6476):405–411. doi:10.1126/science.aax0249

11.	Trapnell C, Cacchiarelli D, Grimsby J, et al. The dynamics and regulators of cell fate decisions are revealed by pseudotemporal ordering of single cells. *Nat Biotechnol*. 2014;32(4):381–386. doi:10.1038/nbt.2859

12.	Lange M, Bergen V, Klein M, et al. CellRank for directed single-cell fate mapping. *Nat Methods*. 2022;19(2):159–170. doi:10.1038/s41592-021-01346-6

13.	Aibar S, González-Blas CB, Moerman T, et al. SCENIC: single-cell regulatory network inference and clustering. *Nat Methods*. 2017;14(11):1083–1086. doi:10.1038/nmeth.4463
